# Factors influencing the outcome of volumetry tools for pulmonary nodule analysis: a systematic review and attempted meta-analysis

**DOI:** 10.1186/s13244-023-01480-z

**Published:** 2023-09-23

**Authors:** Erique Guedes Pinto, Diana Penha, Sofia Ravara, Colin Monaghan, Bruno Hochhegger, Edson Marchiori, Luís Taborda-Barata, Klaus Irion

**Affiliations:** 1 R. Marquês de Ávila E Bolama, Universidade da Beira Interior Faculdade de Ciências da Saúde, 6201-001 Covilhã, Portugal; 2 Liverpool Heart and Chest Hospital NHS Foundation Trust, Thomas Dr, Liverpool, L14 3PE UK; 3 University of Florida, Gainesville, FL 32611 USA; 4 Faculdade de Medicina, Universidade Federal Do Rio de Janeiro, Bloco K - Av. Carlos Chagas Filho, 373 - 2º Andar, Sala 49 - Cidade Universitária da Universidade Federal Do Rio de Janeiro, Rio de Janeiro - RJ, 21044-020 Brasil; 5 Faculdade de Medicina, Universidade Federal Fluminense, Av. Marquês Do Paraná, 303 - Centro, Niterói - RJ, 24220-000 Brasil; 6 Manchester University NHS Foundation Trust, Manchester Royal Infirmary, Oxford Rd, Manchester, M13 9WL UK

**Keywords:** Systematic review, Screening, Cancer, Lung cancer, Computed tomography, Spiral

## Abstract

**Abstract:**

Health systems worldwide are implementing lung cancer screening programmes to identify early-stage lung cancer and maximise patient survival. Volumetry is recommended for follow-up of pulmonary nodules and outperforms other measurement methods. However, volumetry is known to be influenced by multiple factors. The objectives of this systematic review (PROSPERO CRD42022370233) are to summarise the current knowledge regarding factors that influence volumetry tools used in the analysis of pulmonary nodules, assess for significant clinical impact, identify gaps in current knowledge and suggest future research. Five databases (Medline, Scopus, Journals@Ovid, Embase and Emcare) were searched on the 21st of September, 2022, and 137 original research studies were included, explicitly testing the potential impact of influencing factors on the outcome of volumetry tools. The summary of these studies is tabulated, and a narrative review is provided. A subset of studies (n = 16) reporting clinical significance were selected, and their results were combined, if appropriate, using meta-analysis. Factors with clinical significance include the segmentation algorithm, quality of the segmentation, slice thickness, the level of inspiration for solid nodules, and the reconstruction algorithm and kernel in subsolid nodules. Although there is a large body of evidence in this field, it is unclear how to apply the results from these studies in clinical practice as most studies do not test for clinical relevance. The meta-analysis did not improve our understanding due to the small number and heterogeneity of studies testing for clinical significance.

**Critical relevance statement:**

Many studies have investigated the influencing factors of pulmonary nodule volumetry, but only 11% of these questioned their clinical relevance in their management. The heterogeneity among these studies presents a challenge in consolidating results and clinical application of the evidence.

**Key points:**

• Factors influencing the volumetry of pulmonary nodules have been extensively investigated.

• Just 11% of studies test clinical significance (wrongly diagnosing growth).

• Nodule size interacts with most other influencing factors (especially for smaller nodules).

• Heterogeneity among studies makes comparison and consolidation of results challenging.

• Future research should focus on clinical applicability, screening, and updated technology.

**Graphical abstract:**

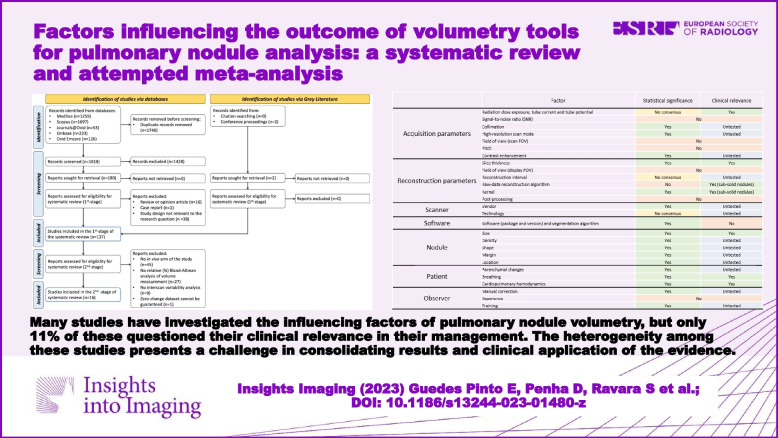

**Supplementary Information:**

The online version contains supplementary material available at 10.1186/s13244-023-01480-z.

## Introduction

Health systems worldwide are implementing Lung Cancer Screening programmes (LCS) to identify early-stage lung cancer and maximise patient survival. However, false positive findings presenting as mostly benign, small, non-calcified pulmonary nodules are present in 22–51% of participants, which may cause morbidity and undermines the cost-effectiveness of LCS [1, 2].

Before the Dutch-Belgian randomised lung cancer screening (NELSON) trial, any pulmonary nodule was considered potentially malignant until proven stable for two years. This trial linked the risk of malignancy to the nodule's size, with small nodules (≤ 100 mm^3^ in volume or ≤ 5 mm in diameter) having a low risk of cancer (0.4%), while large nodules (> 300 mm^3^ or > 10 mm) see this risk raise to 16.9%. The risk of malignancy for medium-sized nodules depends on their growth rate, increasing from 0.8% for nodules with a volume doubling time (VDT) ≥ 600 days to 9.9% for nodules with a VDT < 400 days [1].

Volumetry has consistently outperformed other methods of measuring pulmonary nodules and has been recommended by several international scientific societies for their follow-up [1, 3, 4]. However, the growth curves based on volumetry are highly variable and influenced by multiple known factors [5, 6]. These influencing factors can be related to the scanner, acquisition (e.g., radiation dose exposure, slice thickness) and reconstruction parameters (e.g., kernel), software package, nodule (e.g., size, shape, location), patient (e.g., breathing, comorbidities) or even to the observer (e.g., experience and training). The consistent use of the same scanner, protocol, and software during the follow-up of a pulmonary nodule reduces measurement variability. Still, it is often impractical, such as in cases of equipment failure, critical software upgrades, or the patient moving house.

The primary objective of this systematic review is to summarise the current knowledge regarding the factors that influence the outcome of volumetry tools dedicated to pulmonary nodules. The secondary objectives are to assess the clinical significance of the evidence, identify gaps in current knowledge and suggest future research.

## Methods

The protocol and search strategy were registered with PROSPERO with the registration number CRD42022370233.

The authors defined the primary and secondary research questions as “What factors influence the outcome of volumetry tools dedicated to pulmonary nodules?” and “What is the clinical significance of their effect?” respectively.

The authors searched the following databases on the 21^st^ of September 2022: MEDLINE, SCOPUS, Journals@Ovid, Embase, and Ovid Emcare, using the query: (((Volume OR Volumetry OR Volumetric) AND (lung OR pulmonary) AND (nodule OR nodules)).

### Eligibility criteria

The inclusion criteria were defined as follows:

- Original research studies using dedicated volumetry tools in solid or part-solid pulmonary nodules.
- Study design explicitly tests the potential impact of influencing factors on these tools' outcomes (i.e., volume, segmentation quality).

The exclusion criteria were defined as follows:

- Case reports reviews, or opinion articles.
- Study design exclusively investigating ground-glass opacities (GGOs), using a dedicated (i.e., less generalisable) segmentation algorithm.

The authors excluded duplicate records using the Rayyan online tool (Perdue University).

### Assessment of methodological quality

The quality of the included studies was assessed independently by two authors (chest radiologists with over five years of experience in LCS) based on the revised Quality Assessment of Diagnostic Accuracy Studies (QUADAS-2), and all disagreement was resolved through discussion with a third chest radiologist. The risk of bias was rated as high, low, or unclear.

### Data extraction

Both authors agreed on the final list of reports and retrieved the respective full articles.

Non-English articles (i.e., Chinese, German) were translated using an online service (www.translated.com).

The authors then screened the complete reference lists of all included articles for additional pertinent entries. Grey literature reports were used to identify potential candidate studies.

The variables collected included: population, nodule features, statistical methodology, influencing factor(s), outcome variable, observed effect(s), interactions between different influencing factors, and the statistical significance of relevant tests.

### Statistical analysis and data presentation

To assess the evidence for clinical significance, we selected all in vivo studies reporting interscan variability using relative Bland–Altman analysis. The variables collected at this stage included: influencing factor(s), systematic bias, Limits of Agreement (LOA), and sub-group analysis. The LOA were deduced from the standard deviation and systematic bias if needed. When appropriate, the authors synthesised LOA and systematic bias from groups of studies using the inverse-variance method with a random-effects model (SPSS v26 [IBM, Armonk, NY, USA]).

The heterogeneity between the primary studies was assessed using the heterogeneity variance (τ^2^) and Forest plots. The Deeks’ funnel plot was planned to determine study asymmetry and potential publication bias if comparing more than ten studies.

Missing values were excluded after an unsuccessful attempt to contact the corresponding author of the primary study.

## Results

**Fig. 1:**
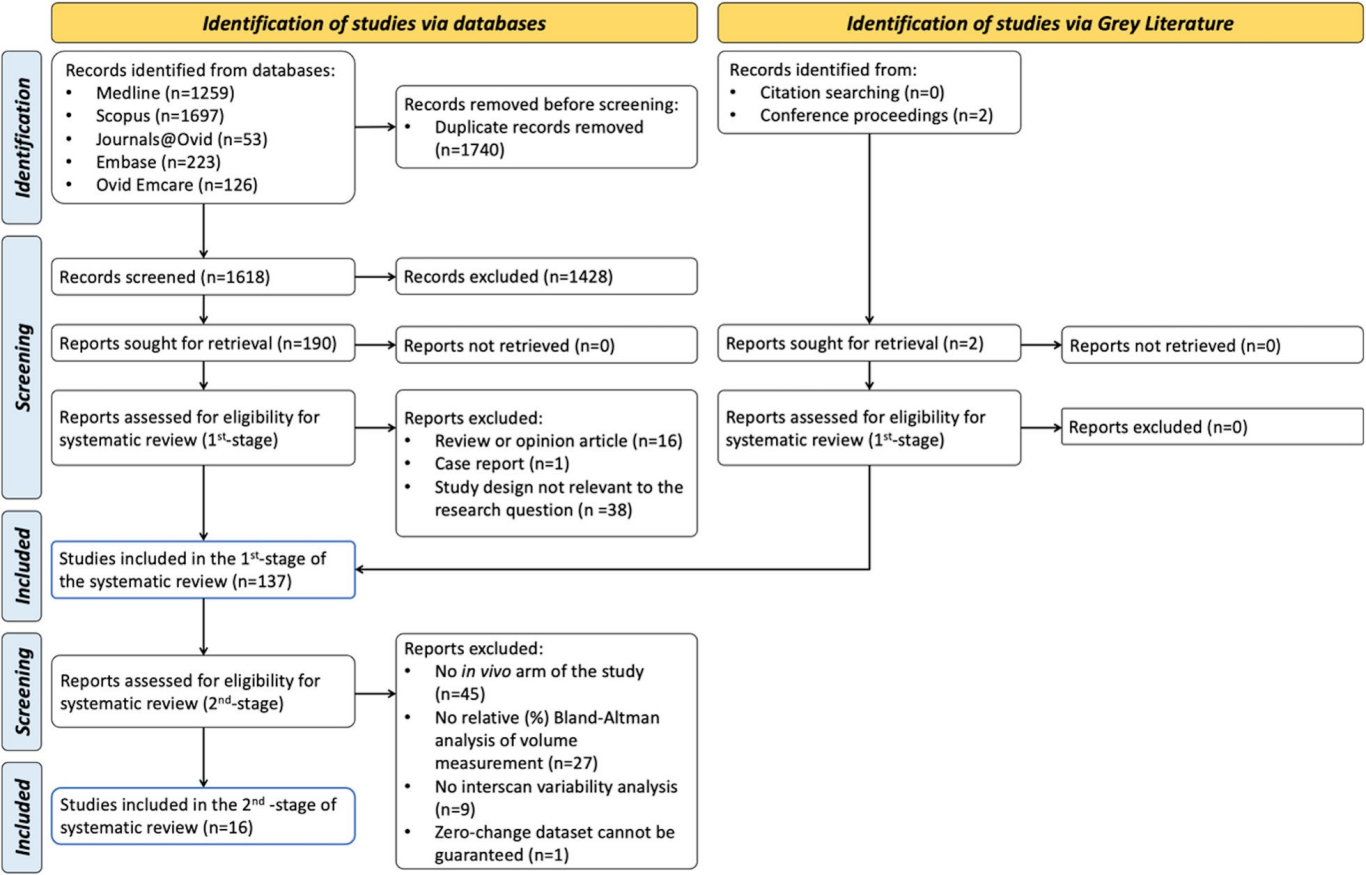
Prisma flow diagram describing the results of the search and selection process

The search returned 1259 (MEDLINE), 1697 (SCOPUS), 53 (Journals@Ovid), 223 (Embase), and 126 (Emcare) results from 1960 to 2022. The PRISMA flow diagram is presented in Fig. 1.

After the study selection and critical appraisal, the first stage of the systematic review included a cohort of 137 studies. A consolidated summary of results is presented in Table 1, and the complete list of the summarised results is provided as Additional file 1: Table S1.

**Table 1:** Summary of studies included in the review

Factor	Statistical significance	Clinical relevance	Observations
*Acquisition parameters*			
Radiation dose exposure, tube current and tube potential	No consensus	Yes	Despite usually considered as non-significant, there are numerous contradictory study results, with some studies even showing inter-scan variability of volumetry measures in the realm of clinical relevance
Signal-to-noise ratio (SNR)	No		Not an independent factor
Collimation	Yes	Untested	Generally considered as clinically not relevant, but untested
High-resolution scan mode	Yes	Untested	Single study showing reduced volume overestimation of pulmonary nodules
Field of view (scan FOV)	No		
Pitch	No		Not significant unless using high pitch mode (pitch factor = 3) in small nodules (< 5 mm)
Contrast enhancement	Yes	Untested	Overestimates the volume of the pulmonary nodule
*Reconstruction parameters*			
Slice thickness	Yes	Yes	Thinner slice thickness improves accuracy, precision, and segmentation quality Should be thin enough to allow any nodule to be visible in ≥ 3 consecutive slices A thickness ≥ 2.5 mm is inadequate to detect 1 mm changes in nodule’s diameter
Field of view (display FOV)	No		
Reconstruction interval	No consensus	Untested	Overlap (interval < thickness) improves accuracy and precision of volumetry in smaller nodules and thicker slices Likely not significant using 1 mm slice thickness
Raw-data reconstruction algorithm	No	Yes (sub-solid nodules)	Iterative reconstruction (IR) algorithms outperform filtered back projection (FBP) for small part-solid nodules and at lower tube currents improving performance of volumetry tools The noise reduction provided by IR is not uniform and less significant at the nodules’ edges
Kernel	Yes	Yes (sub-solid nodules)	Sharp kernel improves volumetry performance in thin 1 mm slices Smooth kernel outperforms sharp kernel in thicker ≥ 2.5 mm slices
Post-processing	No		Image compression and vessel suppression considered as not significantly influencing volumetry of pulmonary nodules
*CT scanner equipment*			
Vendor	Yes	Untested	Only for small nodules not requiring follow-up
Technology	No consensus	Untested	Multi-detector CT, flat-panel, dual energy spectral CT
*Software*			
Software (package and version) and segmentation algorithm	Yes	No	The same software package and version should be consistently used through the follow-up of any pulmonary nodule
*Nodule*			
Size	Yes	Yes	Performance of volumetry tools is degraded in smaller nodules and considered unreliable for growth estimation of nodules < 5 mm
Density	Yes	Untested	Volumetry of non-solid nodules has worse accuracy and precision than for solid nodules
Shape	Yes	Untested	Volumetry of nodules with irregular and spiculated shapes has lower accuracy and precision than volumetry of nodules with round, elongated, smooth or lobulated shapes
Margin	Yes	Untested	Volumetry of nodules with poorly defined margins have higher variability
Location	Yes	Untested	Attachment to surrounding structures (e.g., pleura, vessels, bronchial walls) degrades the performance of volumetry tools
*Patient*			
Parenchymal changes	Yes	Untested	Only with increased attenuation of surrounding parenchyma (e.g., ILD)
Breathing	Yes	Yes	Breathing artifacts are related to volume overestimation and increased measurement variability
Cardiopulmonary haemodynamics	Yes	Yes	Complex cardiopulmonary interactions affecting the amount of blood inside or around a nodule, leading to increased volume measurement variability
*Observer*			
Manual correction	Yes	Untested	Selectively correcting obvious segmentation errors improves the performance of volumetry tools
Experience	No		
Training	Yes	Untested	Training with the volumetry tool is important in unexperienced observers

The second stage of the review identified a cohort of 16 studies, summarising their results in Table 2. Meta-analysis was attempted in two study groups, with results presented as Additional file 1 (Table S3 and Figures S1 and S2). Funnel plots were not performed since the minimum of 10 studies was unmet.

**Table 2:** Summary of studies reporting percent Bland–Altman analysis of interscan variability

Ref.	Population	(n)	Independent variable/subgroup	Bias	_lower_LOA	_upper_LOA
[7]	Patients with known pulmonary nodules					
		100	Size: all	− 0,90%	− 16,40%	14,60%
		58	Size: 30–< 80 mm^3^	− 0.3%	− 16.8%	16.2%
		42	Size: 80–150 mm^3^	− 1.7%	− 15.5%	12.3%
[8]	Patients with pulmonary nodules detected on CCTA	195	Cardiac cycle phase (systole vs diastole)	2.65%	− 47.0%	52.3%
[9]	Patients with part− solid nodules	66	Kernel			
			Solid component segmentation	− 3.2%	− 45.0%	39.0%
			Whole nodule segmentation	13,00%	− 21.0%	46.0%
[10]	Patients under surveillance for < 2 mm solid nodules		Radiation dose exposure (LDCT vs. ULDCT)			
		170	all nodules	− 2.0%	− 18.0%	22.7%
		97	indeterminate nodules	− 6.0%	− 12.7%	21.9%
		68	BMI < 25	− 2.5%	− 17.5%	23.6%
		102	BMI > 25	− 1.0%	− 18.3%	20.8%
[11]	Patients with preoperative scans for subsolid nodules	66	Reconstruction algorithm: FBP vs. MBIR			
			solid component segmentation	6.3%	− 51.9%	64.6%
			whole nodule segmentation	3.2%	− 20.5%	27,00%
[12]	Patients with emphysema	88	Level of inspiration (end-inspiratory vs end-expiratory)	7,5%	− 24,1%	39,1%
[13]	Patients were enrolled prospectively	105	Radiation dose (LDCT vs. ULDCT with FBP or SAFIRE)			
			FBP	0.2%	− 20.0%	20.4%
			SAFIRE	0.3%	− 9.7%	10.4%
[14]	Patients with subsolid nodules	94	intraobserver (R1)	− 1,5%	− 17,3%	16,5%
			Intraobserver (R2)	0,4%	− 14,8%	18,5%
[15]	Patients retrospectively enrolled	202	Radiation dose exposure (SDCT vs. ULDCT)			
			Intraobserver (R1)	1.4%	− 25.1%	26.2%
			Intraobserver (R2)	1.9%	− 25.1%	28.9%
			Interobserver (R1 vs R2)	1.2%	− 25,0%	27.4%
			interobserver (R2 vs R1)	2.1%	− 23.9%	28.1%
[16]	Consecutive patients referred for known or suspected pulmonary metastases (3.3 mm—30 mm)	89	Software			
			Software A	0,0%	− 17,0%	17,0%
			Software B	0,0%	− 13,1%	13,1%
			Software C	0,0%	− 20,8%	20,8%
			Software D	0,0%	− 13,4%	13,4%
			Software E	0,0%	− 20,5%	20,5%
			Software F	0,0%	− 19,6%	19,6%
[17]	Patients on follow-up for lung cancer or scanned because of suspicious pulmonary nodules		Radiation dose exposure (SDCT vs. ULDCT)			
		229	Size: all			
			Intraobserver (R1)	1.5%	− 25.1%	28.1%
			Intraobserver (R2)	2,0%	− 26.4%	30.4%
			Interobserver (R1 vs R2)	1.3%	− 26.5%	29.1%
			interobserver (R2 vs R1)	2.2%	− 25.2%	29.6%
		153	size: < 10 mm			
			Intraobserver (R1)	2.3%	− 28.5%	33.1%
			Intraobserver (R2)	2.6%	− 29.4%	34.6%
			Interobserver (R1 vs R2)	1.9%	− 28.3%	32.1%
			Interobserver (R2 vs R1)	2.1%	− 29,10%	33.3%
		76	Size: ≥ 10 mm			
			Intraobserver (R1)	1.4%	− 18.6%	21.4%
			Intraobserver (R2)	0.4%	− 18.6%	19.4%
			Interobserver (R1 vs R2)	0.4%	− 17,00%	17.8%
			Interobserver (R2 vs R1)	0.6%	− 18.4%	19.6%
[18]	Patients with known nodules were prospectively enrolled	83	Radiation dose: SDCT vs. LDCT			
			SDCT	12.8%	− 27.0%	40.0%
			LDCT	17.0%	− 38.0%	60.0%
[19]	Patients with contrast-enhanced chest CT	101	Slice thickness: 1 mm	− 0.1%	− 21.6%	20.3%
		101	Slice thickness: 3 mm	1.0%	− 15.4%	15.2%
		101	Slice thickness: 5 mm	1.6%	− 21.8%	27.6%
[20]	Patients with pulmonary metastases	218	Segmentation: all	1.3%	− 21.2%	23.8%
		106	Segmentation: complete	0.28%	− 11.9%	12.4%
		112	Segmentation: incomplete	1.61%	− 26.8%	30.0%
[21]	Patients with pulmonary metastases	96	Segmentation algorithm	0.0%	− 26.9%	26.9%
[22]	Patients with pulmonary metastases	151	Size: all	0.7%	− 20.4%	21.9%
		105	Size: < 10 mm	0.55%	− 19.3%	20.4%

The independent variable is the influencing factor (if any) that changes between measurements of each nodule (e.g., standard dose [SDCT], low-dose CT [LDCT] vs. ultra-low-dose CT [ULDCT])

### Influencing factors related to the scanner

#### Acquisition parameters

*Radiation dose exposure, tube current, and tube potential* Minimising radiation dose exposure is essential to LCS and can be done by manipulating tube current and potential, often interchangeably. The interaction between dose exposure and tube current–time product (mAs) is linear and well understood. However, the interaction with tube potential is not, with a reduction of tube voltage from 100 to 80kVp resulting in a reduction of dose exposure in the order of 1.5 [23].

Several studies investigated the impact of radiation dose exposure, tube voltage, or tube current on the outcome of volumetry tools. Less than half of the studies showed a statistically significant difference in accuracy, and the vast majority concluded this difference to be clinically insignificant [13, 15, 24–38]. Some studies reported worsening segmentation quality with lower dose exposure [30] and reduced precision with lower dose exposure, tube current–time product, or tube voltage, limited to small 5 mm and non-solid nodules [28, 39–46].

The impact of radiation dose exposure on volumetry showed clinically significant differences between standard-dose (SDCT) vs low-dose (LDCT) [18] CT protocols and SDCT vs ultra-low-dose CT protocols (ULDCT) [15, 17], contradicting the consensus that reducing the radiation dose does not affect the outcome of volumetry. Studies comparing LDCT vs ULDCT did not confirm this result, thus supporting their use in LCS [10, 13]. Despite the acceptance of SDCT, LDCT and ULDCT protocols, their definition varies among authors, and the effective radiation dose depends on the patient’s body weight. The estimated effective dose acceptable for LCS is 2 mSv [47].

The signal-to-noise ratio (SNR) is not an independent influencing factor [32, 48].

#### Collimation

The effect of collimation is statistically significant between thin (≤ 0.75 mm) and thick (≥ 1.5 mm) settings, with some authors recommending thinner [37, 38] while others recommend thicker [49, 50] settings for volumetry. However, the consensus considers collimation as not clinically significant.

#### High-resolution scan mode

The development of garnet detectors in CT scanners enabled the high-resolution scan mode, increasing the sampling per gantry rotation, spatial resolution, and image quality while reducing volume overestimation [51].

#### Field-of-view (FOV)

The scanners’ spatial resolution in the axial plane depends on the FOV and the matrix size. The scan FOV determines the amount of raw data acquired, but images can be later reconstructed with a different and smaller display FOV.

Several authors investigated the effect of changing the FOV (between 9.6 cm and 36 cm) and showed no statistically significant impact on volumetry [52–55].

#### Pitch

Likewise, the pitch parameter has no significant impact on volumetry within conventionally used values [36, 49, 53, 56], apart from improved repeatability with smaller pitch values (0.9 vs 1.2) [49]. However, the high pitch mode (i.e., pitch factor of 3) reduces the accuracy of volumetry in small (< 5 mm) solid nodules [56].

#### Contrast enhancement

Contrast enhancement overestimates the volume, possibly by increasing the attenuation of the nodules or adjacent structures [57–61]. Rampinelli et al. found volumetry comparable across different delay times (i.e., phases) in contrast-enhanced CT [58].

### Reconstruction parameters

#### Slice thickness

Slice thickness has been investigated as an influencing factor of volumetry between 0.625 and 5 mm. Thinner slices resulted in statistically significant improvement in accuracy and precision in all but one study [19, 21, 31, 34, 36, 39, 44, 48–50, 52–55, 62–67]. In comparison, thicker slices are related to lower measurement agreement and reduced segmentation quality [52, 54, 66].

The slice thickness determines the scan's longitudinal (z-axis) spatial resolution. The difference between the higher axial and lower longitudinal spatial resolution explains why the FOV is insignificant while the slice thickness is, especially for thicker slices.

Increasing the slice thickness increases the volume of voxels along the z-axis. Larger voxels may increase the volume measurement, but surface voxels will also suffer more partial volume effects, increasing measurement variability [54]. Since smaller nodules have a higher ratio of surface to inner voxels, the volumetry of smaller nodules is more affected by slice thickness [21, 36, 49, 52].

The slice thickness should be thin enough to make any nodule visible in at least three consecutive slices [52]. Likewise, a thickness ≥ 2.5 mm is inadequate to detect 1 mm changes in diameter [63, 65].

#### Reconstruction interval

When the reconstruction interval is smaller than the slice thickness (i.e., overlap), the longitudinal spatial resolution improves independently of slice thickness [36, 55].

In a study by Gavrielides et al., the accuracy and precision of volumetry tools improved with 50% overlap, with significant cross-effects between reconstruction interval, slice thickness, nodule size, and radiation dose exposure [36]. Honda et al. reported that non-overlapping reconstructions were associated with volume overestimation in scans obtained with 2.5 mm and 3.75 mm slice thicknesses [54]. Eberhard et al. found no significant benefit of overlapping protocols when using a 1 mm slice thickness, arguing in favour of skipping them to improve LCS cost-effectiveness [68].

#### Raw-data reconstruction algorithm and kernel

CT image reconstruction involves converting the raw data to a sinogram (representing the number and angulation of photons as they hit the detectors) and then to a matrix of attenuation values, known as the image model. This process is called direct back-projection and results in significant blurring. In filtered back projection (FBP), filters (or kernels) are applied to the image model to reduce the blurring effect, provide smoothing or edge enhancement, and highlight certain features and anatomical components.

Most studies investigating the impact of kernels on volumetry have considered them statistically significant (10 out of 13 studies). High-spatial frequency (sharp) kernels, like lung or bone, improved accuracy, precision, and repeatability in most studies [36, 45, 49, 63]. In contrast, a single study reported increased repeatability with a low-spatial frequency (smooth) kernel [64]. Larici et al. investigated the interaction between kernel and slice thickness to conclude that a sharp kernel provides the best performance for volumetry in 1.25 mm slice thickness. A smooth kernel outperforms the sharp kernel in 2.5 mm slice thickness [66].

Several studies reported an overestimation of volume associated with the sharp kernel [54, 59, 64], especially in non-overlapping acquisition and solid nodules (or solid components of part-solid nodules) [54]. Conversely, volumetry of GGOs (or ground-glass components of part-solid nodules) results in higher estimates when using a smooth kernel [9].

In iterative reconstruction (IR), the scanner converts the image model into an artificial sinogram (forward projection). It then compares it to the original sinogram with each iteration, correcting random fluctuations in photon measurement. This process minimises noise and improves image quality at significantly lower radiation exposure [69]. However, this noise reduction is less significant at the edges of the pulmonary nodules, resulting in IR-specific measurement error for small nodules and lower doses or higher noise levels [25, 34].

Multiple studies investigated the influence of raw data reconstruction algorithms on volumetry tools [13, 24, 25, 28, 30, 32–34, 39–42, 51, 70–72], with the consensus being that IR outperforms FBP for small, part-solid nodules or at lower tube currents [28, 39–41], allowing IR-based protocols to replace FBP safely.

Recently, Kim et al. [73] investigated two deep learning (DL)-based raw-data reconstruction algorithms (Truefidelity and ClariCT.AI), showing improved accuracy against the adaptive statistical iterative reconstruction (ASiR) algorithm using LDCT and ULDCT.

The scientific literature often refers to raw-data reconstruction algorithms and kernels as just reconstruction algorithms, which could be confusing since the former is generally considered not to influence volumetry measurements. At the same time, the latter is known to do so [42].

#### Post-processing

Despite the earlier warning by Ko et al. regarding image compression [74], Santos et al. found no significant deterioration in the performance of volumetry tools within the limits proposed in the European Society of Radiology (ESR) position paper [75, 76].

The influence of vessel suppression on volumetry was investigated by Milanese et al. using commercially available software (ClearRead, Riverain, Miamisburg, OH, USA). The authors reported high measurement agreement with and without vessel suppression, although the rate of manual correction was unusually high (49/77, 75.4%) [77].

### CT scanner equipment

#### CT scanner vendor

Comparing the performance of volumetry tools using different scanners showed good accuracy regardless of the scanner vendor [37]. Two later studies found a statistically significant difference between scanner vendors, but only for small nodules, which would not require follow-up according to current guidelines [26, 78].

#### CT scanner technology

Several studies have compared the performance of volumetry between different scanner technologies (e.g., single or multiple detectors, flat-panel, and dual-energy CT scanners) [19, 37, 55, 79, 80]. Das et al. reported increasing accuracy in volumetry with more detector rows [38], although Xie et al. did not confirm this [81].

Flat-panel scanners outperform multi-detector scanners in pulmonary nodule volumetry, especially in small nodules (< 5 mm) [82–85].

Mono-energetic reconstructions at 70 keV using dual-energy spectral CT are considered equivalent to conventional CT images acquired using 120 kVp, and several authors found no significant difference in volumetry accuracy between them [86–88]. In addition, mono-energetic reconstructions improved the repeatability of volumetry at the same radiation dose [87].

### Influencing factors related to the software

#### Software package and segmentation algorithm

Several studies compared different software packages and different segmentation algorithms for pulmonary nodule volumetry, reporting statistically significant differences in all but one study [45, 83, 89–92] and even between different versions of the same software [93]. Adjusting the attenuation threshold, as some segmentation algorithms allow, also influences the volume measurement outcome [45, 55, 92].

Several international societies firmly recommend consistently using the same software package, version, and segmentation algorithm during follow-up [94].

### Influencing factors related to the nodule

#### Nodule size

Volumetry is less performant for small nodules [10, 13, 16, 18, 21, 25, 28, 29, 31, 36–38, 40, 41, 45, 48–51, 53, 55, 56, 60, 63, 70, 72, 74, 78, 79, 81, 82, 89, 92, 93, 95–101], explained mainly by partial volume effects, and is considered unreliable for nodules < 5 mm in diameter [60, 102].

Multiple interactions between nodule size and other influencing factors are known, including collimation [31, 49], tube current [29, 41], reconstruction algorithm [29, 41, 51, 70, 72], kernel [36], reconstruction interval [36], slice thickness [20, 21, 31, 36, 48, 49, 53], scanner technology [41, 52, 82], software [16, 45, 89, 93], compression level [74], density [28, 41], and level of inspiration [16, 20].

Hwang et al. suggested that raising the threshold to 9 mm for starting follow-up would lead to a significant increase in specificity (i.e., from 91.7% to 96.7%) at the cost of only a modest decrease in sensitivity (i.e., from 96.2% to 94.2%). The impact of such a change to current recommendations would result in a 60% reduction of follow-up scans at the cost of delaying the diagnosis of 1.9% of lung cancer patients [103]. Volumetry tools should be robust to influencing factors for solid nodules ≥ 9 mm when using current LDCT protocols in LCS programmes.

#### Density

Published studies in the literature describe the density of a nodule as either a qualitative (e.g., solid, part-solid, ground-glass, calcified) or quantitative feature (i.e., in Hounsfield Units).

Non-solid nodules are more challenging to segment manually and using volumetry tools and present lower accuracy and higher variability than solid nodules [11, 25, 26, 28, 36, 41, 45, 92].

Interactions between density and other influencing factors have been described, including nodule size [28, 36, 92], reconstruction algorithms [28, 41, 70], slice thickness [36], tube current [41], level of inspiration [104], and image compression [74]. Higher nodule density is correlated to larger volume [88].

#### Shape and margin

The shape of a pulmonary nodule can be round, elongated, smooth, lobulated, spiculated, or irregular.

An irregular or spiculated shape is associated with lower accuracy [62, 64] and precision [20] of volumetry tools. It is also associated with a lower volume measurement [78], lower segmentation quality [97, 105], and increased variability [21, 96, 97, 100, 106].

The ratio of surface to inner voxels increases in nodules with an irregular or spiculated shape (i.e., larger surface area), deteriorating the performance of volumetry tools due to partial volume effects [78, 100]. Therefore, volumetry of small (≤ 6 mm) pulmonary nodules with irregular or spiculated shapes (i.e., high-risk features for malignancy) may be unreliable and can justify an optional follow-up period [107].

The shape of a nodule also interacts with other influencing factors, such as the nodule's density [28], location, slice thickness, and kernel [64].

Several authors describe spiculation as a feature of the nodule's margin, which can be a source of confusion. We defined the margin as either well or poorly defined. In a study by Iwano et al., volume measurements of nodules with poorly defined margins had a significantly higher variability [108].

#### Location

Most authors categorise a nodule’s location as either intra-parenchymal, juxta-pleural, juxta-fissural, or juxta-vascular [37, 38, 51, 64, 66, 96, 109, 110], with intra-parenchymal nodules further classified as either central or peripheric [74, 111, 112].

Attachments to adjacent structures (e.g., vessels, bronchial wall, and pleura) may result in the latter's inclusion, overestimating the volume and increasing the measurement variability [111, 112].

In a recent study by Guedes Pinto et al., the authors reported the location in both the axial (anterior, middle, or posterior) and coronal (upper, middle, lower) planes, additionally measuring the vascular distance along the pulmonary arteries, from the main pulmonary artery (MPA) to the nodule using multiplanar reformatting, which proved to be statistically significant [113]. Conversely, the location within a lobe [18] or segment [98] was not proven to be statistically significant.

Interactions have been reported between the location and software [111], shape [64], slice thickness [64, 66], kernel [64], tube current [66], and compression [74].

### Influencing factors related to the patient

#### Parenchymal changes

Both global and regional parenchymal changes in emphysema patients (i.e., reduced parenchymal attenuation) have been investigated and found not significantly to affect pulmonary nodule volumetry (108. However, in diseases with increased parenchyma attenuation, like interstitial lung disease (ILD), the reduced contrast between nodule and surrounding parenchyma could deteriorate the performance of volumetry tools. In two phantom studies by Gavrielides et al., the difference in attenuation between a synthetic nodule and the background was statistically significant [39, 67]. Recently, Penha et al. reported that the quality of pulmonary nodule segmentation by volumetry tools decreases with increasing attenuation of the surrounding parenchyma [114].

#### Breathing

Breathing artefacts are related to overestimating volume and increased measurement variability of volumetry tools [12, 16, 20, 43, 99, 104, 115, 116]. This effect is most significant at the end of expiration and for smaller nodules but is considered unlikely to be clinically relevant [12, 16, 20, 115]. However, Goo et al. reported a volume overestimation of 23.1% from inspiration to expiration, interpreted as clinically significant [116].

The level of inspiration interacts with other influencing factors like the nodule size [16], density [104], and software package [16].

#### Cardiopulmonary haemodynamic factors

Studies designed with coronary CT angiography (CCTA) can compare the performance of volumetry tools at different cardiac phases in a single acquisition.

Boll et al. reported changes in volume measurement related to a complex interaction between the cardiac phase, location (i.e., pulmonary segments), and nodule size [98].

Guedes Pinto et al. investigated the impact of cardiopulmonary haemodynamic factors on volumetry tools, including the cardiac phase, calibre change of the MPA between systole and diastole, the vascular distance between the MPA and the nodule, and nodule's location along the axial (related to hydrostatic pressure) and coronal plane (related to vascular section area), all statistically significant except the cardiac phase. The authors proposed a theoretical model where the volume of a given nodule is affected by the dynamic vascular pressure as blood travels from the heart to the nodule [113]. In another study by the same authors, the variability of volumetry vastly exceeded the criterion for clinical significance when comparing measurements in opposing cardiac phases (systole vs diastole [− 47%, 52.3%]), with the lower variability seen when comparing two measurements in diastole ([− 18.9%, 19.7%]) [8].

CCTA is not appropriate for LCS. However, there is considerable overlap in risk factors between coronary artery disease and lung cancer. Patients enrolled in LCS are also at risk of cardiovascular events, with some authors advocating a role for dual screening [113].

### Influencing factors related to the observer

#### Manual correction, observer experience and training

The promise of (semi)automated tools is to reduce interobserver variability by limiting the observer's influence in the measurement [102, 117]. Counter-intuitively, allowing manual correction of the segmentation improves the tool’s performance [60, 102, 118]. This is explained because inadequately segmented nodules tend to be outliers (i.e., either including adjacent structures [113] or incompletely segmenting the nodule [20]), resulting in higher variability and lower observer agreement.

The outcome of volumetry tools is independent of observer experience (i.e., radiologists vs non-radiologists), even when manually correcting the segmentation result. However, in the un-experienced group of observers, training with the tool was statistically significant for volume measurements [119].

### Regarding concerns of bias and excluded studies

The most common concern of bias in the included studies (Table 3) is the use of experimental algorithms [9, 28, 45, 53, 74, 89, 92, 117, 120–152], followed by the assumption of zero-change datasets over more extended periods, relying on the perceived stability of the nodules [80, 95, 152]. Two studies use non-consecutive or convenience sample techniques, possibly introducing selection bias [60, 71]. Still, others present an incomplete description of their methods, poorly defining their population or the statistical analysis [26, 72, 77, 93, 148, 152].

**Table 3:** Assessment of bias of the primary studies

Specific concerns of bias	References
Experimental algorithm not commercially available	[9, 28, 45, 53, 74, 89, 92, 117, 120–152]
Assumption of zero-change dataset cannot be guaranteed	[80, 95, 152]
Inadequate description of statistical analysis	[72, 93, 148, 152]
Non-consecutive or convenience sample	[60, 71]
Study population is inadequately described	[26, 77]

Several promising candidate studies were excluded after full-text analysis based on their choice of outcome (Additional file 1: Table S2). These outcomes include the risk of malignancy [1, 100, 103, 153–161], prognosis [162–167], growth [5, 67, 101, 112, 168–174], or comparison to other methods of measurement like diameter [100, 175], area [175], the diameter of an equivalent volume sphere [3] or manual segmentations (e.g., most of the recent research using DL-based segmentation).

Although these outcomes are clinically interesting, they are unrelated to our research questions.

## Discussion

The influencing factors of volumetry tools have been investigated extensively. However, the possibility of wrongly diagnosing a nodule as stable or growing between follow-up scans has only been tested in a little over 10% of studies. Consolidating the results from different studies is difficult due to the heterogeneity, but an impact on clinical decision-making seems more likely in smaller nodules.

The contrast between nodule and surrounding lung parenchyma and the surface-to-inner voxel ratio are two key concepts in understanding how volumetry tools can be influenced.

Pulmonary nodule volumetry benefits from the contrast between the nodule and the surrounding well-aerated lung parenchyma. This contrast is decreased in sub-solid nodules when the surrounding parenchyma has increased attenuation (e.g., ILD, expiratory phase, contrast enhancement) or when the nodule contacts adjacent structures. Image reconstruction with different kernels and raw-data reconstruction algorithms may also expand or contract the segmentation by changing the attenuation value of the voxels.

Surface voxels contain both nodule tissue and surrounding parenchyma and suffer partial volume effects leading to measurement error and variability.

The surface-to-inner voxel ratio depends primarily on the size difference between the nodule and the voxel (i.e., how many voxels fit in the nodule). Still, it can also be increased by an irregular shape or ill-defined nodule margin (i.e., increased surface area).

Reducing the slice thickness and measuring nodules of increasing size rapidly decreases the ratio of surface to inner voxels, improving the performance of volumetry tools.

Apart from these two key concepts, implementation details involved in the segmentation algorithms account for most of the remaining observed influence in volumetry tools.

Despite the large number of included studies in this review, comparing study results is problematic given a large number of influencing factors and heterogeneity in study design, outcomes, statistical analysis, nodule features and demographics. Additionally, multiple authors report statistically significant results while openly questioning their clinical relevance. Changing a factor that influences a volumetry tool may not be enough to change our assessment of nodule growth and clinical management. Therefore, using this evidence to support clinical decisions is challenging. We consider this a limitation of the evidence and a strong motivator for this review.

A clarification of clinical significance seems needed. The optimal waiting period for a follow-up scan is based on the inherent in vivo interscan measurement variability of volumetry tools, accepted as ≤ 25% of total volume [6]. Higher measurement variability implies a longer time to distinguish real growth from measurement error. Therefore, we defined clinical significance as interscan variability > 25% of volume change since false-positive growth estimation would become more likely in this setting. We used this criterion to select a subset of all studies reporting interscan variability using Bland–Altman analysis (n = 16). Influencing factors investigated regarding their clinical relevance include radiation dose exposure, slice thickness, raw-data reconstruction algorithms, kernels, size, cardiac cycle phase, software package, segmentation algorithm, and level of inspiration.

We combined the results of two studies comparing SDCT vs ULDCT protocols [15, 17], and the synthesised result confirmed the primary studies’ conclusions. We also combined the results in a second group (three studies) by disregarding sub-group analysis concerning size [7, 22] and quality of segmentation [20], with a synthesised result within the clinically acceptable a priori LOA, but losing the influence of the factors (i.e., size and quality of segmentation) under study. Due to significant population, outcome, and design heterogeneity, we could not combine other studies. Therefore, our attempted meta-analysis failed to advance the current knowledge meaningfully (Additional file 1: Table S3 and Figures S1 and S2).

Several other factors have been statistically shown to influence the outcome of volumetry tools. However, the clinical relevance of these findings still needs to be investigated (Table 1) and represents gaps in current knowledge and opportunities for future research.

### Implications of the results for practice, policy, and future research

Findings from this review confirm the clinically significant impact of some known influencing factors on pulmonary nodule volumetry, including the segmentation algorithm, quality of the segmentation, slice thickness, the level of inspiration for solid nodules, and the reconstruction algorithm and kernel in subsolid nodules (Table 3).

Much of the evidence collected has yet to be tested for potential clinical significance and is thus open for future research.

A concern related to this systematic review is the long period of the included studies in a rapidly changing field, suggesting that this review may not reflect current performance. A comparison of recent (i.e., last five years) and older studies show an improving performance trend likely related to software and scanner technology innovations. In a recent study by Bartlett et al., the reported interscan variability was not clinically relevant (_95_CI [− 16.8%; 16%]) even for very small (30–80 mm^3^) solid, non-metastatic and non-calcified pulmonary nodules (n = 58), suggesting that a shorter optimal waiting time may already be appropriate [7].

We propose a standard for future studies around the Bland–Altman analysis and restricted to nodules between 5 and 10 mm where growth estimation is useful. Such studies should investigate the persisting gaps in current knowledge, focusing on clinical applicability and currently available technology. Future research should also explore the cost and benefits of potential changes to current practices, like raising the threshold for follow-up or shortening the optimal waiting period in the follow-up schedule.

### Supplementary Information

**Additional file 1: Supplementary Table S1. **Studies included in the first stage of the systematic review [176–182]. **Supplementary Table S2. **Studies that seemed promising for inclusion during the screening phase but were later excluded [183, 184]. **Supplementary Table S3. **Results of the attempted meta-analysis of two sets of similar studies. **Supplementary Figures S1. **Forest plot of similar studies [15, 17]. **Supplementary Figures S2. **Forest plot of similar studies [7, 20, 22].

## Data Availability

All data extracted or synthesised and the analytic code used for the meta-analysis can be obtained from the corresponding author on reasonable request.

## Abbreviations

CCTA: Coronary CT angiography
DL: Deep-learning
FBP: Filtered back-projection
FOV: Field of view
GGO: Ground-glass opacity
IR: Iterative reconstruction
LCS: Lung cancer screening
LDCT: Low-dose CT
LOA: Limits of agreement
QUADAS-2: Quality Assessment of Diagnostic Accuracy Studies tool
SDCT: Standard dose CT
SNR: Signal-to-noise ratio
ULDCT: Ultra-low-dose CT
VDT: Volume doubling time
