# Supplementary material for: Factors influencing the outcome of volumetry tools for pulmonary nodule analysis: a systematic review and attempted meta-analysis
Source: Insights Imaging. 2023 Sep 23;14:152. doi: 10.1186/s13244-023-01480-z (PMC10517915; doi:10.1186/s13244-023-01480-z)
Supplement: Supplementary file 1 — Additional file 1: Supplementary Table S1. Studies included in the first stage of the systematic review [176–182]. Supplementary Table S2. Studies that seemed promising for inclusion during the screening phase but were later excluded [183, 184]. Supplementary Table S3. Results of the attempted meta-analysis of two sets of similar studies. Supplementary Figures S1. Forest plot of similar studies [15, 17]. Supplementary Figures S2. Forest plot of similar studies [7, 20, 22]. [file 13244_2023_1480_MOESM1_ESM.docx]

**Factors influencing the outcome of volumetry tools for pulmonary nodule analysis: a systematic review and attempted meta-analysis**

**ELECTRONIC SUPPLEMENTARY MATERIAL**

**Supplementary Table 1:** Studies included in the first stage of the systematic review

| ref | Authors | Year | (n) | population | CT protocol | statistical methodology | outcome | Influencing factor understudy | Effect | statistical significance | | | interactions |  |  |
| --- | --- | --- | --- | --- | --- | --- | --- | --- | --- | --- | --- | --- | --- | --- | --- |
| (7) | Agnes et al. | 2022 | 1010 | CT data from open access *in vivo* databases (LIDC) | SDCT | Similarity analysis | Dice coefficient | segmentation algorithm  (UNet vs GAP CNN) | ↑ | |  |  | |  |  |
| (8) | Bartlett et al. | 2022 | 100 | Patients with small (30 - 150mm^3^) non-metastatic pulmonary nodules | 128CT | Bland-Altman (mm3, %) | Interscan variability |  | [-14.2mm^3^, 12mm^3^]  [-16.4%, 14.6%] | |  |  | |  |  |
| (9) | Debnath et al. | 2022 | 888 | CT data from open access *in vivo* databases (LUNA-16) | SDCT | Similarity analysis | Dice coefficient | segmentation algorithm (MF-3D UNet vs custom UNets) | ↑ | |  |  | |  |  |
| (10) | Lu et al. | 2022 | 1018 | CT data from open access *in vivo* databases (LIDC-IDRI) | SDCT | Similarity analysis | Dice coefficient,  Precision, recall | segmentation algorithm (Dense-UNet vs FCN32s, SegNet, UNet, BN-UNet) | ↑ | |  |  | |  |  |
| (11) | Kido et al. | 2022 | 332 | Local patient database | SDCT | Similarity analysis | Dice coefficient,  Intersection-Over-Union | segmentation algorithm (3-D fully connected CNN vs 3D U-Net, 3D SegNet, watershed, graphcut) | ↑ | |  |  | |  |  |
| (12) | Pinto et al. | 2022 | 195 | Patients with pulmonary nodules detected on CCTA | CCTA | Bland-Altman (%) | Interscan variability | Cardiac phase (systole, diastole) | [-47.0%, 52.3%] | |  |  | |  |  |
|  |  |  |  |  |  |  |  | Inter-software | [-50.2%, 68.2%] | |  |  | |  |  |
|  |  |  |  |  |  |  |  | Inter-observer | [-14.5%, 27.8%] | |  |  | |  |  |
| (13) | Pinto et al. | 2022 | 195 | Patients with pulmonary nodules detected on CCTA | CCTA | Multiple linear regression | volume | cardiac phase (systole, diastole) |  | | N |  | |  |  |
|  |  |  |  |  |  |  |  | vascular distance from MPA to nodule | ↓ | | Y |  | |  |  |
|  |  |  |  |  |  |  |  | MPA caliber change from systole to diastole | ↑ | | Y |  | |  |  |
|  |  |  |  |  |  |  |  | location | ↑ (lower third, posterior third) | | Y |  | |  |  |
|  |  |  |  |  |  |  |  | adequacy of segmentation (inadequate, adequate) | ↓ | | Y |  | |  |  |
| (14) | Song et al. | 2022 | 1018 | CT data from open access *in vivo* databases (LIDC) | SDCT | Similarity analysis | Dice coefficient | segmentation algorithm(LNHG vs UNet, nnUNet, L-MSE, L-VGG) |  | |  |  | |  |  |
|  |  |  | 91 | Local patient dataset |  |  |  | (solid LNHG vs UNet) | ↓ | |  |  | |  |  |
|  |  |  |  |  |  |  |  | (juxta-pleural LNHG vs UNet) | ↓ | |  |  | |  |  |
|  |  |  |  |  |  |  |  | (juxta-vascular LNHG vs UNet) | ↓ | |  |  | |  |  |
|  |  |  |  |  |  |  |  | (solid LNHG vs others) | ↑ | |  |  | |  |  |
|  |  |  |  |  |  |  |  | (juxta-pleural LNHG vs others) | ↑ | |  |  | |  |  |
|  |  |  |  |  |  |  |  | (juxta-vascular LNHG vs others) | ↑ | |  |  | |  |  |
| (15) | Werner et al. | 2022 | 54 | Patients with part-solid pulmonary nodules | SDCT | Bland-Altman (%) | intraobserver variability |  | solid component [-70%,49%] whole PSN [-111%,31%] | |  |  | |  |  |
|  |  |  |  |  |  |  | interobserver variability |  | solid component [-16%,16%] whole PSN [-102%,65%] | |  |  | |  |  |
|  |  |  |  |  |  |  | interscan variability | kernel (smooth, hard) | solid component [-45.0%,39%] whole PSN [-21.0%,46%] | |  |  | |  |  |
| (16) | Lu et al | 2022 | 55 | Local patient dataset | SDCT | Similarity analysis | Dice coefficient | kernel (lung, mediastinal, bone) |  | | N |  | |  |  |
| (17) | Bianconi et al. | 2021 | 111 | Local *in vivo* database PET/CT | SDCT | Similarity analysis | Dice coefficient | segmentation algorithm (DL-based vs non-DL based) |  | |  |  | |  |  |
|  |  |  | 100 | CT data from open access *in vivo* databases (LIDC-IDRI) |  |  |  |  |  | |  |  | |  |  |
| (18) | Cui et al. | 2021 | 192 | NSCLC patients | SDCT (DVN) | Similarity analysis | Dice coefficient | ﻿density (solid, part-solid, GGO) | ↓ | |  |  | |  |  |
| (19) | Dutande et al. | 2021 | 1010 | CT data from open access *in vivo* databases (LIDC, LNDb, ILCID) | SDCT | Similarity analysis | Dice coefficient | segmentation algorithm (SquExUNet vs 3D NodNet) | ↑ | |  |  | |  |  |
|  |  |  | 294 |  |  |  |  |  |  |  |  |  | |  |  |
|  |  |  | 113 |  |  |  |  |  |  |  |  |  | |  |  |
| (20) | Yu et al. | 2021 | 1010 | CT data from open access *in vivo* databases (LIDC) | SDCT | Similarity analysis | Dice coefficient | segmentation algorithm (3D-ResNet, 3D-UNet) | ↑ | |  |  | |  |  |
| (21) | Jain et al. | 2021 | 100 | CT data from open access *in vivo* databases (LIDC-IDRI) | SDCT | Similarity analysis | Dice coefficient, Jaccard index | segmentation algorithm (SSSOA-based GAN vs kernel-based Bayesian clustering, SVM+K-nearest, FCM, region-based segmentation, DDRN, CoLe-CNN) | ↑ | |  |  | |  |  |
| (22) | Meng et al. | 2021 | 1018 | CT data from open access *in vivo* databases (LIDC-IDRI) | SDCT | Similarity analysis | Dice coefficient | ﻿kernel (lung, mediastinal, bone) |  | | N |  | |  |  |
| (23) | Kim et al. | 2021 | 4+3 | solid and ground-glass artificial nodules | LD-CT vs ULD-CT | RM-ANOVA | Accuracy (APE) | reconstruction algorithm (DL-based: Truefidelity, ClariCT.AI, non-DL-based: ASiR) | ↑(↓) | | Y |  | |  |  |
|  |  |  |  |  |  |  |  | (for all density, size and radiation exposure) |  |  |  |  | |  |  |
| (24) | Shi et al. | 2021 | 58 | Local patient dataset | SDCT | Similarity analysis | Accuracy | ﻿segmentation algorithm (multiscale residual UNet vs fuzzy C-means clustering) | ↑ | |  |  | |  |  |
| (25) | Wang et al. | 2021 | 1010 | CT data from open access *in vivo* databases (LIDC) | SDCT | Similarity analysis | Dice coefficient | segmentation algorithm (MGSANet vs SegNet, FCN8s, UNet, UNet++) | ↑ | |  |  | |  |  |
| (26) | Wang et al. | 2021 | 847 | CT data from open access *in vivo* databases (LIDC-IDRI) | SDCT | Similarity analysis | Dice coefficient, Jaccard index | segmentation algorithm (H-DL vs deep Unet, shallow UNet) | ↑ | |  |  | |  |  |
| (27) | Zhang et al. | 2021 | 1018 | CT data from open access *in vivo* databases (LIDC-IDRI) | SDCT | Similarity analysis | Dice coefficient, Jaccard index | ﻿segmentation algorithm (UNet with BN + ⍺-hull vs UNet with BN, UNet with ⍺-hull | ↑ | |  |  | |  |  |
| (28) | Dong et al. | 2020 | 874 | CT data from open access *in vivo* databases (LIDC-IDRI) | SDCT | Similarity analysis | ﻿DICE, ASD, HSD, accuracy, sensitivity | ﻿segmentation algorithm(MV-SIR vs MV-CNN, MV-I-CNN) | ↑ | |  |  | |  |  |
| (29) | He et al. | 2020 | 10 | 5 solid + 5 GGO synthetic nodules | dual-energy CT | Wilcoxon signed-rank | Accuracy (APE) | SECT vs DECT for same radiation dose exposure | ↑(↓) | | Y |  | |  |  |
| (30) | Lee et al. | 2020 | 8 | spherical synthetic nodules (diameter 5, 8, 10, 12m; attenuation +100HU, -630HU) | 256CT | Wilcoxon signed-rank Generalized estimating equations (GEE) | Accuracy (APE) | helical vs axial scan |  | | N |  | |  |  |
|  |  |  |  |  |  |  |  | reconstruction algorithm - FBP vs ASIR (30%, 50%) |  | | N |  | |  |  |
|  |  |  |  |  |  |  |  | tube current (10, 20, 30, 100mAs) |  | | N |  | |  |  |
|  |  |  |  |  |  |  |  | density | ↑(↓) | | Y |  | |  |  |
|  |  |  |  |  |  |  |  | nodule size | ↑(↓) | | Y |  | |  |  |
| (31) | Penha et al. | 2020 | 1265 | LCS participants | LDCT | Binary logistic regression | Adequate segmentation | attenuation of adjacent lung parenchyma | ↓ | | Y |  | |  |  |
| (32) | Rakshith et al | 2020 | 888 | CT data from open access *in vivo* databases (LUNA-16) | SDCT | Similarity analysis | Dice coefficient | segmentation algorithm (segmentation network of 2D-CNNs vs UNet, R2UNet, SUMNet) | ↑ | |  |  | |  |  |
| (33) | Usman et al. | 2020 | 1010 | CT data from open access *in vivo* databases (LIDC-IDRI) | SDCT | Similarity analysis | Dice coefficient, HD, sensitivity, PPV | segmentation algorithm (A-ROI with multi-view deep residual learning vs constant ROI with multi-view deep residual learning) | ↑ | |  |  | |  |  |
| (34) | Wu et al. | 2020 | 936 | CT data from open access *in vivo* databases (LIDC-IDRI) | SDCT | Similarity analysis | Dice coefficient | segmentation algorithm (3D-UNet + training 3D-CRF vs 2D-UNet, 2D-UNet-CRF, 3D-UNet, 3D-UNet-CRF) | ↑ | |  |  | |  |  |
| (35) | Aresta et al. | 2019 | 1012 | CT data from open access *in vivo* databases (LIDC-IDRI) | SDCT | Similarity analysis | Intersection-Over-Union | Segmentation algorithm (iW-Net vs 3D-UNet) | ↑ | |  |  | |  |  |
|  |  |  |  |  |  |  |  |  |  | |  |  | |  |  |
| (36) | Eberhard et al. | 2019 | 66 | synthetic nodules (diameter: 4 - 10mm; density: 20-80HU) | SDCT, LDCT, ULDCT, DECT | ANOVA | Accuracy (APE) | radiation dose exposure (1/8th, 1/20th, 1/70th of standard dose) | ↓(↑) (at 1/70th) | | Y |  | |  |  |
|  |  |  |  |  |  |  |  | reconstruction algorithm (FBP, ADMIRE) | ↓(↑) (ADMIRE at strength level 5) | | Y |  | |  |  |
|  |  |  |  |  |  |  |  | nodule diameter | ↑(↓) | | Y |  | |  |  |
|  |  |  |  |  |  |  |  | density | ≠ | | Y |  | |  |  |
| (37) | Kim et al. | 2019 | 8 | synthetic nodules (diameter: 3, 5, 7, 9mm; density: 50HU); PSN (outer GGO diameter: 20mm; density: -650HU; inner solid portion similar to solid nodule) | 128CT | repeated-measures ANOVA | Accuracy (APE) | radiation dose exposure (120/100, 120/50, 120/20, 120/10, 80kVp/10mAs) | ↓(↑) | | Y |  | |  |  |
|  |  |  |  |  |  |  |  |  |  | |  |  |  |  |  |
|  |  |  |  |  |  |  |  | reconstruction algorithm (FBP, HIR, MBIR) | ↑(↓) (MBIR) | | Y |  |  |  |  |
|  |  |  |  |  |  |  |  | density | ↑(↓) | | Y |  |  |  |  |
| (38) | Lui et al. | 2019 | 986 | CT data from open access *in vivo* databases (LIDC-IDRI) | SDCT | Similarity analysis | ﻿Dice coefficient, ASD, sensitivity, PPV | ﻿segmentation algorithm (CDP-ResNet + IWS, CDP-ResNet + RS, CDP-ResNet + WS) | ↑ | |  |  | |  |  |
| (39) | Liu et al. | 2019 | 11 | 6 solid + 5 GGO synthetic nodules | 256CT | mixed analysis of variance on aligned ranks | Accuracy (APE) | tube voltage (80, 100, 120kV) | ↑(↓) | | Y | density * tube voltage | |  |  |
|  |  |  |  |  |  |  |  | tube current (10 to 30 mAs) |  | | N |  |  |  |  |
|  |  |  |  |  |  |  |  | density | ↑(↓) | | Y |  |  |  |  |
| (40) | Balagurunathan et al. | 2018 | 100 | LCS participants | LDCT | Concordance correlation coefficient | volume | tube current (10, 20, 30, 100mAs) |  | | N |  | |  |  |
|  |  |  |  |  |  |  | volume variability | density | ↑(↓) | | Y |  |  |  |  |
| (41) | Chung et al. | 2018 | 84 | Local patient dataset | SDCT | Similarity analysis | ﻿Dice coefficient, MHD, sensitivity, specificity and accuracy | ﻿segmentation algorithm (active contour model and baysian approach vs Chan-vese, normalized and modified Chan-Vese, snake algorithm) | ↑ | |  |  | |  |  |
| (42) | Gavrielides et al. | 2018 | 8 | synthetic nodules (diameter: 5 - 5.75, 8 - 8.75mm) phantom (background difference: 1000, 50-100HU) | 16CT | AUROC | percent error, CV | nodule to background contrast (low, high) | ↓ | | Y |  | |  |  |
| (43) | Jin et al. | 2018 | 82 | Patients with cancer | LDCT, SDCT,  256CT | paired sample t-test | volume | nodule size | ↑(↓) | | Y |  | |  |  |
|  |  |  |  |  |  |  |  |  | (part solid, calcified) | | N |  | |  |  |
| (44) | Kakinuma et al. | 2018 | 226 | Patients with stable nodules | 16CT | Bland-Altman (%) | intraobserver variability |  | [-33.5%,26.5%] | |  |  | |  |  |
|  |  |  |  |  |  |  | interobserver variability |  | [-17.1%,67.9%] | |  |  | |  |  |
| (45) | Kidera et al. | 2018 | 330 | Local patient dataset | SDCT | Similarity analysis | Dice coefficient, Jaccard index | segmentation algorithm (Conv-DeconvNet vs watershed) | ↑ | |  |  | |  |  |
| (46) | Kim et al. | 2018 | 15120 | simulated virtual sub-solid nodules (diameter: 8, 10, 12mm; contrast to background: +100HU) + (diameter: 6, 8, 10mm; contrast to background: 270HU) | dual-layer CT 64CT | RC | Repeatability | single energy vs monoenergetic images | ↓ | | Y |  | |  |  |
|  |  |  |  |  |  |  |  | tube current (30, 15, 10mAs) | ↑ (less significant with IR) | | Y |  | |  |  |
|  |  |  |  |  |  |  |  | reconstruction algorithm (FBP, IR) | ↓ | | Y |  | |  |  |
| (47) | Milanese et al. | 2018 | 65 | Patients | LDCT | Bland-Altman (mm3) | volume | post-processing (normal, vessel-suppression) |  | | N |  | |  |  |
| (48) | Paks et al. | 2018 | 170 | Patients under LDCT surveillance for solid pulmonary nodules >2mm | LDCT vs ULDCT 128CT | Bland-Altman (%) | interscan variability | radiation dose exposure (LDCT vs ULDCT) | [-18.0%,22.7%] | |  |  | |  |  |
|  |  |  | 97 | subset of indeterminate nodules |  |  |  |  | [-12.7%,21.9%] | |  |  | |  |  |
|  |  |  | 68 | BMI <25 |  |  |  |  | [-17.5%,23.6%] | |  |  | |  |  |
|  |  |  | 102 | BMI ≥25 |  |  |  |  | [-18.3‰,20.8%] | |  |  | |  |  |
| (49) | Qin et al. | 2018 | 1018 | CT data from open access *in vivo* databases (LIDC-IDRI) | SDCT | Similarity analysis | Dice coefficient, PPV, Accuracy | ﻿segmentation algorithm (3D-CNN vs SegNMaps, SegNEdgem Seg-NLBP, Seg-Nsynthetic) | ↑ | |  |  | |  |  |
| (50) | Shakir et al. | 2018 | 72 | CT data from open access *in vivo* databases (LIDC) and phantom databases (CUMC, FDA) | CT | Bland-Altman (mm3) | volume | segmentation method | hybrid method [-0.1,0.3] Gao et al. method [-0.8,1.1] Chan Vese method [-0.1,0.7] | |  |  | |  |  |
| (51) | Cohen et al. | 2017 | 73 | Patients with part-solid pulmonary nodules | SDCT | Bland-Altman | intraobserver variability | reconstruction algorithm (FBP, MBIR) | FBP; solid component [-81.3%,90.8%] FBP, whole SSN [-40.1%,42.6%] MBIR, solid component [-85.8%,74%] MBIR, whole SSN [-35.6%,36.2%] | | N |  | |  |  |
|  |  |  |  |  |  |  | interobserver variability | reconstruction algorithm (FBP, MBIR) | FBP; solid component [-126.2%,129.4%] FBP, whole SSN [-32.6%,51.7%] MBIR, solid component [-142.2%,116.8%] MBIR, whole SSN [-36.3%,47.3%] | | N |  | |  |  |
|  |  |  |  |  |  |  | interscan variability | reconstruction algorithm (FBP, MBIR) | solid component [-51.9%,64.6%] whole SSN [-20.9%,27.3%] | | Y |  | |  |  |
| (52) | Feng et al. | 2017 | 1010 | CT data from open access *in vivo* databases (LIDC) | SDCT | Similarity analysis | Dice coefficient | segmentation algorithm  (UNet vs GAP CNN) | ↑ | |  |  | |  |  |
| (53) | Gavrielides et al. | 2017 | 12 | synthetic nodules (diameter: 5, 10mm; density: -800, -630, -10HU; shape: spherical, spiculated) | LDCT | ANOVA | Accuracy (APE) | density (-800, -630, -10HU) | ↑(↓) | | Y | nodule size * density, nodule size * shape | |  |  |
|  |  |  |  |  |  |  |  | shape (spherical, spiculated) | ↓(↑) | | Y |  |  |  |  |
|  |  |  |  |  |  |  |  | reconstruction algorithm (FBP, AIDR 3D, FIRST) | ≠ | | Y |  |  |  |  |
|  |  |  |  |  |  |  | Repeatability coefficient (RC%) | radiation dose exposure | ↓ (5mm) | | Y |  |  |  |  |
| (54) | den Harder et al. | 2017 | 63 | Consecutive series of patients with non-calcified solid nodules | dual-layer CT | Bland-Altman (mm3, %) | Volume | VNC (40, 70, 100, 130, 160, 200 keV) | ↓ (except at 70keV) | | Y | | | |  |
|  |  |  |  |  |  |  |  | nodule size (≤200mm3, >200mm3) |  | | N |  | | | |
| (55) | Han et al. | 2017 | 100 | LCS participants | LDCT | Bland-Altman (%) | interobserver variability |  | all [-23.7%,23.7%] smooth [-21.4%,21.4%] lobulated [-18.1%,18.1%] spiculated [-28.2%,28.2%] irregular [-27.0%,27.0%] | |  |  | | | |
| (56) | Liang et al. | 2017 | 171 | 117 LCS participants with stable nodules >2 years | 64CT | Levene test | volume variance | software | ≠ | | Y |  | | | |
|  |  |  |  |  |  |  |  | nodule size (<4, 4-5, 6-9, >10mm) | ↓ | | Y |  | | | |
| (57) | Moser et al. | 2017 | 88 | Patients with emphysema | 128CT | Bland-Altman (%) | Volume | Inspiration vs expiration | ↑ [-24.1,39.1%] | | Y |  | |  |  |
|  |  |  |  |  |  | Multiple linear regression | Volume variability | % reduction in lung volume at expiration |  | | N |  | |  |  |
|  |  |  |  |  |  |  |  | local emphysema extent |  | | N |  | |  |  |
|  |  |  |  |  |  |  |  | nodule size |  | | N |  | |  |  |
| (58) | Su et al. | 2017 | 9 | synthetic nodules (diameter: 2.5, 5, 10mm; density: -100HU, 60HU, 100HU) | 64CT | repeated-measures ANOVA | Accuracy (APE) | tube current (10, 20, 50, 80, 100, 150, 350mAs) |  | | N |  | |  |  |
|  |  |  |  |  |  |  |  | reconstruction algorithms (FBP, ASIR 30%, ASIR 50%, ASIR 80%) |  | | N |  |  |  |  |
|  |  |  |  |  |  |  |  | nodule diameter | ↑(↓) | | Y |  |  |  |  |
| (59) | Talwar et al. | 2017 | 40 | Patients with indeterminate pulmonary nodules (20) + lung metastases (20) | LDCT | Bland-Altman (%) | interscan variability |  | [-22.6%; 35.6%] | |  |  | | | |
|  |  |  |  |  |  | Mann-Whitney U test | volume | level of inspiration |  | | N |  | | | |
|  |  |  |  |  |  |  | volume variability | Nodule size | slightly smaller for nodules <500mm3 | |  |  | | | |
|  |  |  |  |  |  |  |  | location |  | | N |  | | | |
| (60) | Wang et al. | 2017 | 847 | CT data from open access *in vivo* databases (LIDC-IDRI) | SDCT | Similarity analysis | Dice coefficient,  ASD | segmentation algorithm (MV-CNN vs level set, graph cut) | ↑ | |  |  | | | |
| (61) | Wang et al. | 2017 | 1018 | CT data from open access *in vivo* databases (LIDC-IDRI) | SDCT | Similarity analysis | ﻿Dice coefficient, ASD, sensitivity, PPV | ﻿segmentation algorithm (CF-CNN vs CF-CNN-MP, level set, graphcut, UNet) | ↑ | |  |  | | | |
|  |  |  | 74 | Local *in vivo* patient dataset |  |  |  |  |  |  |  |  | | | |
| (62) | Kalpathy-Cramer et al. | 2016 | 52 | *In vivo* clinical databases (CIA) | SDCT | two-way ANOVA | bias | segmentation algorithms | ≠ | | Y |  | | | |
|  |  |  | 12 | phantom database (CUMC) |  | Concordance correlation coefficient | volume repeatability |  | ≠ | | Y |  | |  |  |
|  |  |  |  |  |  |  | volume reproducibility |  | ≠ | | Y |  | |  |  |
| (63) | Li et al. | 2016 | 157 | *In vivo* clinical database from several hospitals + open access database (LIDC) | SDCT | Jaccard index | Adequacy of segmentation (error rate) |  | juxtavascular 0.17 ground-glass opacities 0.2 juxtavascular GGO 0.24 | |  |  | | | |
| (64) | Ohno et al. | 2016 | 30 | simulated nodules (diameter: 3, 5, 8, 10, 12mm, density: -800, -630, +100HU) | LDCT | ANOVA | Accuracy (APE) | reconstruction algorithm (FBP, AIDR 3D, FIRST) | ↑(↓) (at 10 and 20mAs) | |  |  | |  |  |
|  |  |  |  |  |  |  |  | tube current (10, 20, 40, 80, 120, 200, 270mAs) | ↑(↓) | |  |  | |  |  |
| (65) | Sui et al. | 2016 | 105 | Patients resembling NLCST | LDCT, ULDCT | Bland-Altman (%) | interobserver variability | reconstruction algorithm (FBP, SAFIRE) | FBP [-12.9%,12.1%] SAFIRE [-12%,13.1%] | |  |  | | | |
|  |  |  |  |  |  |  | interscan variability | radiation dose exposure (LDCT vs ULDCT) | FBP [-20%,20.4%] SAFIRE [-9.7%,10.4%] | |  |  | | | |
| (66) | den Harder et al. | 2016 | 37 | Patients under pulmonary nodule follow-up | 256CT | Wilcoxon signed-rank test | volume | tube current (60, 33, 24, 15 mAs) | ↓ | | Y |  | | | |
|  |  |  |  |  |  |  |  | reconstruction algorithm (FBP vs hybrid vs MBIR) | ↓ (MBIR) | | Y |  | | | |
| (67) | Gavrielides et al. | 2015 | 8 | synthetic nodules (outer and inner diameters: 20/10, 10mm/5mm; densities: 100/-630, 10/-630, -630/100, -630HU/-10HU) | 16CT | ANOVA | Accuracy (APE) | collimation (0.75,1.5) |  | | Y | nodule size * collimation x slice thickness, collimation x slice thickness * radiation dose exposure | | | |
|  |  |  |  |  |  |  |  | slice thickness (0.8,1.5,2,3,5mm) |  | | Y |  |  |  |  |
|  |  |  |  |  |  |  |  | kernel (medium, hard) |  | | N |  |  |  |  |
|  |  |  |  |  |  |  |  | tube current (20, 100, 200mAs) |  | | N |  |  |  |  |
| (68) | Hwang et al. | 2015 | 45 | synthetic nodules (diameter: 4, 6, 8mm; density: 90HU) | dual-energy 128CT | paired t-test | Accuracy (RPE) | pitch (conventional = 1, high-pitch = 3) | ↓(↑) | | Y |  | |  |  |
|  |  |  |  |  |  | Tuckey's test | IVV | nodule size | ↓ | | Y |  |  |  |  |
| (69) | Lassen et al. | 2015 | 59 | *In vivo* open access database of SSN | CT | Jaccard index Wilcoxon signed-rank test | agreement with manual reference segmentation | segmentation algorithm |  | |  |  | | | |
| (70) | Li et al. | 2015 | 48 | synthetic nodules (diameter: 5, 8, 10, 20mm; shape: spherical, elliptical, lobulated, spiculated); density: -630, -10, +100 HU) | 16CT | Linear regression, analysis of variance and restricted maximum likelihood | volume variability | nodule size (5, 8, 10, 20mm) | ↓ | | Y | nodule size * slice thickness x collimation, nodule size * shape, nodule size * density, shape * density | |  |  |
|  |  |  |  |  |  |  |  | slice thickness x collimation (0.8x0.75, 1.5x0.75, 3x0.75, 2x1.5, 3x1.5, 5x1.5mm) | ↑ | | Y |  |  |  |  |
|  |  |  |  |  |  |  |  | radiation dose exposure |  | | N |  |  |  |  |
|  |  |  |  |  |  |  |  | kernel (detail, medium) | ↑ | | Y |  |  |  |  |
|  |  |  |  |  |  |  |  | attachment to vessels or chest wall | ↑ | | Y |  |  |  |  |
|  |  |  |  |  |  |  |  | pitch (0.9, 1.2) | ↑ | | Y |  |  |  |  |
| (71) | Young et al. | 2015 | 33 | Patients with clinical indication to follow a pulmonary nodule | SDCT | paired t-test | intraobserver variability repeatability reproducibility | simulated dose reduction (25%, 10, 3%) |  | | N |  | | | |
|  |  |  |  |  |  |  |  | reconstruction algorithm (FBP, SAFIRE) |  | | N |  | | | |
| (72) | Christie et al. | 2014 | 113 | Patients with pulmonary nodules >3mm, not attached to vessels or pleura | 64CT | Bland-Altman | interscan variability | software (LMS Lung, LungCare) | [-53.9%,138.4%] | | Y |  | | | |
|  |  |  | 13 |  |  |  |  | kernel (b30, b70) | [-7.9%,49%] | | Y |  | | | |
|  |  |  |  |  |  |  |  | nodule size |  | | N |  | | | |
| (73) | Doo et al. | 2014 | 8 | synthetic nodules (diameter: 5, 8, 10, 12mm); density: +100HU, -630HU | LDCT | Linear mixed model regression | Accuracy (APE) | tube current (10, 20, 30, 50 mAs) | ↑(↓) | | Y | nodule size*tube current, density*tube current, density*scan type, nodule size*density, nodule size* scan type | |  |  |
|  |  |  |  |  |  |  |  | reconstruction algorithm (FBP vs IR) | ↑(↓) | | Y |  |  |  |  |
|  |  |  |  |  |  |  |  | scan type (helical vs volumetric) | ↑(↓) | | Y |  |  |  |  |
|  |  |  |  |  |  |  |  | nodule size | ↑(↓) | | Y |  |  |  |  |
|  |  |  |  |  |  |  |  | density | ↑(↓) | | Y |  |  |  |  |
| (74) | Guo et al. | 2014 | 86 | Patients with non-calcified pulmonary nodules 3-30mm | 64CT | Logistic regression | Segmentation adequacy | shape (smooth, lobulated, irregular) | ↓ | | Y |  | | | |
|  |  |  |  |  |  |  |  | location (intra-parenchymal, attached to vessel, attached to pleura) | ↓ (attached to vessel or pleural) | | Y |  | | | |
|  |  |  |  |  |  | Bland-Altman | interscan variability |  | [-14%,-11.6%] | |  |  | | | |
| (75) | Kim et al. | 2014 | 6 | synthetic nodules (diameter: 10, 12mm); density: +100, -630, -800HU) | 256CT | Generalizes estimating equations | Accuracy (APE) | radiation dose exposure |  | | N |  | |  |  |
|  |  |  |  |  |  |  |  | reconstruction algorithm (FBP, IR) |  | | N |  | |  |  |
|  |  |  |  |  |  |  |  | density | ↑(↓) | | Y |  | |  |  |
| (76) | Koike et al. | 2014 | 71 | Patients with peripheral lung cancer ≤30mm | 16CT, 64CT | Bland-Altman (%) | interobserver variability |  | [-18.6%; 15.4%] | |  |  | | | |
| (77) | Petrick et al. | 2014 | 10 | synthetic nodules (diameter: 10, 20mm; shape: spherical, elliptical, lobulated, spiculated; density: -10, +100HU) | 16CT | ANOVA | volume bias | slice thickness (0.8, 5mm) | ↓ | | Y |  | |  |  |
|  |  |  |  |  |  |  | volume variability | slice thickness (0.8, 5mm) | ↓ | | Y |  | |  |  |
| (78) | Zhao et al. | 2014 | 147 | LCS participants | 16CT | Wilcoxon signed-rank test | volume | software (LungCare, OncoTreat, Vitrea) | ≠ | | Y |  | | | |
|  |  |  |  |  |  |  |  | location (peripheral, non-peripheral) |  | | Y |  |  |  |  |
|  |  |  |  |  |  |  |  | attachment (intra-parenchymal, fissure attached, vessel attached, pleural attached) |  | | Y |  |  |  |  |
|  |  |  |  |  |  |  |  | shape (spherical, non-spherical) |  | | Y |  |  |  |  |
|  |  |  |  |  |  |  |  | margin (smooth, nonsmooth) |  | | Y |  |  |  |  |
| (79) | Chen et al. | 2013 | 35 | synthetic nodules (diameter: 4.76, 9.53mm; density: 80HU | dual-energy CT | Generalized estimating equations | Accuracy (APE) | reconstruction algorithm (FBP, ASIR, MBIR) | ≠ (larger nodules) | | Y |  | |  |  |
|  |  |  |  |  |  |  |  | slice thickness (0.625,1.25,2.5) | ↑(↓) | | Y |  | |  |  |
|  |  |  |  |  |  |  |  | software (Lung VCAR, iNtuitioin) | ≠ | | Y |  | |  |  |
|  |  |  |  |  |  |  | volume variability | reconstruction algorithm (FBP, ASIR, MBIR) |  | | N |  | |  |  |
|  |  |  |  |  |  |  |  | slice thickness (0.625,1.25,2.5) |  | | N |  | |  |  |
|  |  |  |  |  |  |  |  | software (Lung VCAR, iNtuitioin) |  | | N |  | |  |  |
| (80) | Christe et al. | 2013 | 579 | synthetic nodules (density: -630, +100HU; diameter: 5, 8, 10, 12mm) | 64CT | Confidence intervals | Accuracy (APE) | radiation dose exposure | ↑(↓) (at the lowest dose level 25mAs/100kVp) | | N |  | |  |  |
|  |  |  |  |  |  |  |  | nodule size at highest dose level (100mAs) | ↓(↑) (10, 12mm) | | Y |  | |  |  |
|  |  |  |  |  |  |  | volume variability |  | ↑ (at lowest dose level) | | Y |  | |  |  |
| (81) | Coenen et al. | 2013 | 4 | synthetic nodules (diameter: 5, 8, 10, 12mm); density: +100HU | 64CT | one-way ANOVA | Accuracy (APE, RPE) | radiation dose exposure | ↑(↓) | | Y |  | |  |  |
|  |  |  |  |  |  |  |  | nodule size (5, 8, 10, 12mm) | ↑(↓) (5mm) | | Y |  | |  |  |
|  |  |  |  |  |  |  |  | scan mode (conventional, high-resolution) | ↑(↓) | | Y |  | |  |  |
|  |  |  |  |  |  |  |  | iterative reconstruction level (0%, 50%, 100%) | ↑(↓) | | N |  | |  |  |
| (82) | Gavrielides et al. | 2013 | 2880 | synthetic nodules (diameter: 5, 8, 10, 20mm; shape: spherical; density: -630, +100HU) | 64CT | n-way ANOVA | volume | overlapping (0%, 50%) | ↓ (smaller nodules) | | Y | overlap * nodule size, overlap * slice thickness, overlap * radiation dose exposure, nodule size * density, nodule size * slice thickness, nodule size * kernel, density * slice thickness | |  |  |
|  |  |  |  |  |  |  |  | slice thickness | ↓ (even more in thicker slices) | | Y |  |  |  |  |
|  |  |  |  |  |  |  |  | density | ↓ | | Y |  |  |  |  |
|  |  |  |  |  |  |  | volume variability | overlapping (0%, 50%) | ↓ (smaller nodules) | | Y |  |  |  |  |
|  |  |  |  |  |  |  |  | slice thickness | ↓ (even more in thicker slices) | | Y |  |  |  |  |
|  |  |  |  |  |  |  |  | density | ↓ | | Y |  |  |  |  |
|  |  |  |  |  |  |  | volume or volume variability | radiation dose exposure |  | | N |  |  |  |  |
|  |  |  |  |  |  |  |  | pitch |  | | N |  |  |  |  |
|  |  |  |  |  |  |  |  | kernel (B40f, B60f) |  | | N |  |  |  |  |
| (83) | Kim et al. | 2013 | 94 | Patients with PSN and GGO | 16CT | Bland-Altman (%) | intraobserver variability | R1 | [-7.6%,8.5%] | |  |  | | | |
|  |  |  |  |  |  |  |  | R2 | [-7.4%,8.5%] | |  |  | | | |
|  |  |  |  |  |  |  | interobserver variability |  | [-11.7%,18.1%] | |  |  | | | |
|  |  |  |  |  |  |  | interscan variability | R1 | [-17.3%,16.5%] | |  |  | | | |
|  |  |  |  |  |  |  |  | R2 | [-14.8%,18.5%] | |  |  | | | |
| (84) | Scholten et al. | 2013 | 33 | LCS participants with SSN >5mm | LDCT 16CT | Bland-Altman (mm3,%) | semi-automatic volume vs manual volume | intra-observer variability | R1 [-72.8%,24.1%] R2 [-89.0%,36.0%] | |  |  | | | |
| (85) | Tan et al. | 2013 | 55 | *In vivo* open-access database (CIA) | 64CT | Descriptive statistics | Volume overlap ratio |  | All 70%  <20mm 69% | |  |  | | | |
|  |  |  |  |  |  | ICC |  |  | 0.998 | |  |  | | | |
|  |  |  | 22 | synthetic nodules (diameter: 10, 20mm; density: -630, +100HU) |  |  | Accuracy (APE) | nodule size | ↑(↓) (smaller nodules) | | Y |  | |  |  |
|  |  |  |  |  |  | 95% Confidence interval |  |  | [-21%,15%] | |  |  | |  |  |
| (86) | Wielputz et al. | 2013 | 162 | synthetic nodules (density 20HU) | MDCT | Repeated measures ANOVA on ranks | Accuracy (APE) | radiation dose exposure | ↑(↓) | |  |  | |  |  |
|  |  |  |  |  |  | Wilcoxon signed-rank | Accuracy (APE) | reconstruction algorithm (FBP vs IR) all dose exposure |  | | N |  | |  |  |
|  |  |  |  |  |  |  | volume | reconstruction algorithm (FBP vs IR) dose exposure < 1mGy | ↓ | | Y |  | |  |  |
| (87) | Willemink et al. | 2013 | 78 | Patients with solid pulmonary nodules and known or suspected malignancy | 64CT | Bland-Altman (mm3) | volume | reconstruction algorithm (FBP vs IR) |  | | N |  | | | |
| (88) | Xie et al. | 2013 | 15 | synthetic nodules (diameter: 3, 5, 8, 10, 12mm; density: -800, -630, +100HU) | 16CT, 64CT, LDCT | Bland-Altman (%) | interobserver variability |  | 14mm3 [-54.3%,34.4%] 65mm3 [-40.7%,49.4%] 268mm3 [-26.2%,22.1%] 524mm3 [-26.2%,22.1%] 905mm3 [-20.3%,19.2%] | |  |  | |  |  |
|  |  |  |  |  |  |  | interscan variability |  | 14mm3 [-55.6%,49.0%] 65mm3 [-35.8%,42.2%] 268mm3 [-24.2%,27.1%] 524mm3 [-23.7%,24.9%] 905mm3 [-18.7%,19.4%] | |  |  | |  |  |
|  |  |  |  |  |  | t-test | volume | nodule size | ↓ | | Y |  | |  |  |
| (89) | Xie et al. | 2013 | 5 | synthetic nodules (diameter: 3, 5, 8, 10, 12mm; density: +100HU) | LDCT (64CT) | Bland-Altman | volume | CT vendor (all sizes) |  | | N |  | |  |  |
|  |  |  |  |  |  |  |  | CT vendor (3mm nodules) |  | | Y |  | |  |  |
|  |  |  |  |  |  |  | interscanner and intrascanner variability | nodule size | ↓ | |  |  | |  |  |
| (90) | Xie et al. | 2013 | 40 | synthetic nodules (shape: irregular, lobulated; density: -51, 2, 57, 125, 157HU) | 64CT | Bland-Altman (%) | interobserver |  | [-33.9%, 40.4%] | |  |  | |  |  |
|  |  |  |  |  |  |  | interscan |  | [-33.3%, 34.5%] | |  |  | |  |  |
|  |  |  |  |  |  | Univariate analysis | volume | CT scanner (Sensation 64 Siemens, Brilliance 64 Philips) | ↓ | | Y |  | |  |  |
|  |  |  |  |  |  |  |  | density (-51, 2, 57, 125, 157HU) |  | | N |  | |  |  |
|  |  |  |  |  |  |  |  | shape (spiculated, lobulated) | ↓ | | Y |  | |  |  |
| (91) | de Jong et al. | 2012 | 101 | Patients with lung metastases | SDCT enhanced, LDCT | Bland-Altman (%) Wilcoxon signed-rank test | volume | protocol (SDCT enhanced, LDCT) | ↓ (<200mm3) | | Y |  | | | |
|  |  |  |  |  |  |  |  |  | (>200mm3) | | N |  | | | |
| (92) | Willemink et al. | 2012 | 5 | synthetic nodules (diameter: 3, 5, 8, 10, 12mm; density: +100HU) | 256CT | Visual assessment | Accuracy (APE) | nodule size | ↑(↓) | | N |  | |  |  |
|  |  |  |  |  |  |  |  | reconstruction algorithm (FBP vs IR) |  | | N |  | |  |  |
|  |  |  |  |  |  |  |  | tube current (25, 50, 100 mAs) | ↑(↓) (size 3mm) | | N |  | |  |  |
|  |  |  |  |  |  |  |  | tube voltage (80, 100, 120kV) | smallest at 100kVp | | N |  | |  |  |
| (93) | Yang et al. | 2012 | 52 | Patients under follow-up for indeterminate pulmonary nodules <15mm | 64CT, SDCT | repeated-measures ANOVA | volume | kernel (standard, bone, lung) | ↑ | | Y |  | | | |
|  |  |  |  |  |  |  |  | slice thickness (0.625, 1.25, 2.5mm) | ↑ (2.5mm) | | Y |  | | | |
| (94) | Ashraf et al. | 2010 | 188 | LCS participants with nodules > 5mm | 16CT | Bland-Altman | interobserver variation | segmentation algorithm (small size, all size, subsolid) | ↓ | | Y |  | | | |
|  |  |  |  |  |  |  | volume | segmentation algorithm (small size, all size, subsolid) | ↑ | | Y |  | | | |
| (95) | Hein et al. | 2010 | 202 | Patients with primary lung carcinoma, lung metastases or suspicious nodules | 16CT | Bland-Altman | interobserver variability | radiation dose exposure (LDCT vs ULDCT) | [-12.6%;12.4%] | |  |  | | | |
|  |  |  |  |  |  |  | Interscan variability |  | [-25.1%;-23.4%] | |  |  | | | |
| (96) | Rampinelli et al. | 2010 | 35 | Patients with pulmonary nodules | 16CT, SDCT | Wilcoxon signed-rank test | volume | contrast enhancement (pre-, post-contrast) | ↑ | | Y |  | | | |
|  |  |  |  |  |  |  |  | contrast delay (30, 60, 120, 180, 300s) |  | | N |  | | | |
|  |  |  |  |  |  |  |  | software | ≠ | | Y |  | | | |
| (97) | Rinaldi | 2010 | 100 | Patients with solid intra-parenchymal pulmonary nodules | 16CT, SDCT | case count | volume variability | segmentation software version (LungCare 2006G, 2007G) |  | |  |  | | | |
| (98) | Wang et al. | 2010 | 200 | LCS participants | 64CT, LDCT | Bland-Altman | volume repeatability | slice thickness (1, 2mm) | ↓ | | Y | shape * location * slice thickness * kernel | | | |
|  |  |  |  |  |  |  |  | kernel (soft, sharp) | ↓ | | Y |  |  |  |  |
| (99) | Iwano et al. | 2009 | 60 | Patients with peripheral lung cancer | 64CT, SDCT | ANOVA | interobserver variability |  |  | | N |  | | | |
|  |  |  |  |  |  |  |  | margins (clear, obscure) | ↓ | | Y |  | | | |
|  |  |  |  |  |  |  |  | nodule size (≤20, >20mm) |  | | N |  | | | |
|  |  |  |  |  |  |  |  | density (solid, part solid, nonsolid) |  | | N |  | | | |
|  |  |  |  |  |  |  |  | spiculation (present, absent) |  | | N |  | | | |
|  |  |  |  |  |  |  |  | attachment to vessels or chest wall (present, absent) |  | | N |  | | | |
| (100) | Marchiano et al. | 2009 | 233 | LCS participants | 16CT | Bland-Altman | volume | interscan variability ±27% |  | |  |  | | | |
| (101) | Marten et al. | 2009 | 31 | synthetic nodules (diameter:2.94 - 10.1mm, shape: spherical, density: 35HU) | fpCT, 64CT | ANOVA | intraobserver variation | scanner type (fpCT, 64CT) | ↑ | | Y |  | |  |  |
|  |  |  |  |  |  |  | interobserver variation | scanner type (fpCT, 64CT) | ↑ | | Y |  |  |  |  |
|  |  |  |  |  |  | Wilcoxon signed-rank test | volume | scanner type (fpCT, 64CT) | ↓ | | Y |  |  |  |  |
|  |  |  |  |  |  |  | volume variability | nodule size | ↓ | | Y |  |  |  |  |
|  |  |  |  |  |  |  |  | slice thickness (0.625, 1.25mm) | ↑ | | Y |  |  |  |  |
|  |  |  |  |  |  |  |  | location |  | | N |  |  |  |  |
| (102) | Nietert et al. | 2009 | 29 | synthetic nodule (diameter: 3 - 15.9mm) | 16CT | Nonlinear regression model | volume variability | slice thickness (0.625, 1.25, 2.5, 5mm) | ↑ | | Y |  | |  |  |
| (103) | Dehmeshki et al. | 2008 | 815 | *In vivo* clinical database | SDCT | case count | Subjectively adequate segmentation result |  | 83 - 85% | |  |  | | | |
|  |  |  |  |  |  |  |  | location | ↓ (juxtavascular) | |  |  |  |  |  |
| (104) | de Hoop et al. | 2008 | 214 | Patients with lung metastases | LDCT | Bland-Altman (upper LOA %) | interscan variability | software | highest with software D and satisfactory segmentation 27.6% | |  |  | | | |
|  |  |  |  |  |  | F-test |  | software | ≠ | | Y |  |  |  |  |
|  |  |  |  |  |  | F-test |  | adequacy of segmentation (excellent, satisfactory) | ↑ | | Y |  |  |  |  |
|  |  |  |  |  |  | one-way ANOVA |  | nodule size | ↓ | | Y |  |  |  |  |
|  |  |  |  |  |  | Pearson correlation coefficient |  | level of inspiration |  | | N |  |  |  |  |
| (105) | Hein et al. | 2008 | 229 | 33 patients: 25 for staging or follow-up; 8 with suspected pulmonary nodules | 16CT | Bland-Altman (%) | intrascan SDCT | radiation dose exposure (SDCT vs ULDCT) | [-8%,9.4%] | |  |  | | | |
|  |  |  |  |  |  |  | intrascan ULDCT |  | [-12.9%,13.1%] | |  |  | | | |
|  |  |  |  |  |  |  | interscan (SDCT vs ULDCT) |  | R1 vs R2 [-26.5%,29.1%] R2 vs R1 [-25.2%,29.6%] R1 vs R1 [-25.1%,28.1%] R2 vs R2 [-26.4%,30.4%] | |  |  | | | |
| (106) | Larici et al. | 2008 | 3 | synthetic nodules (location: juxtavascular, intraparenchymal, juxtapleural; density: +100HU) | 16CT | Student t-test | Accuracy (difference cm3) | tube current (40, 100mAs) |  | | N |  | |  |  |
|  |  |  |  |  |  |  |  | tube current (40, 100mAs) with lung kernel | ↑(↓) | | Y |  | |  |  |
|  |  |  |  |  |  |  |  | slice thickness (1.25, 2.5mm) |  | | N |  | |  |  |
|  |  |  |  |  |  |  |  | slice thickness (1.25, 2.5mm) with 40mAs and high-spatial kernel | ↓(↑) | | Y |  | |  |  |
|  |  |  |  |  |  |  |  | kernel (lung, detail, bone, bone+) | lung kernel most accurate for 100mAs and 2.5mm slice thickness bone+ kernel most accurate for 40mAs and 1.25mm slice thickness | |  |  | |  |  |
|  |  |  |  |  |  |  | successful segmentation | location (juxtavascular, intraparenchymal, juxtapleural) | juxtapleural nodule, 2.5mm slice thickness and 40mAs failed to segment | |  |  | |  |  |
|  |  |  |  |  |  |  |  | kernel (lung, detail, bone, bone+) | only lung kernel segmented the juxtapleural nodule | |  |  | |  |  |
| (107) | Tateishi et al. | 2008 | 44 | 3 patients with lung metastases | 4D 256CT | one-way ANOVA | volume | level of inspiration | ↑ (end-expiratory) | | Y |  | | | |
| (108) | Rampinelli et al. | 2008 | 83 | Patients with lung nodules between 5-10mm | LDCT, SDCT | Bland-Altman (%) | interscan SDCT |  | [-19.7%,53.7%] | |  |  | | | |
|  |  |  |  |  |  |  | interscan LDCR |  | [-9.7%,35.3%] | |  |  | | | |
|  |  |  |  |  |  | Spearman correlation coefficient |  | % emphysema |  | | N |  | | | |
|  |  |  |  |  |  |  |  | nodule size |  | | N |  | | | |
|  |  |  |  |  |  | Kruskal-Wallis test |  | shape (smooth, lobulated, irregular) |  | | N |  | | | |
|  |  |  |  |  |  |  |  | location |  | | N |  | | | |
| (109) | Ravenel et al. | 2008 | 56 | synthetic nodules (diameter: 3 - 16mm; shape: spherical, lobulated) | 16CT | General linear regression | Accuracy (APE) | kernel (standard, lung, bone, bone+, edge, detail, soft) |  | | N |  | |  |  |
|  |  |  |  |  |  |  |  | slice thickness (0.625, 1.25, 2.5, 5mm) | ↓(↑) | | Y |  |  |  |  |
|  |  |  |  |  |  |  |  | nodule size | ↑(↓) | | Y |  |  |  |  |
|  |  |  |  |  |  |  |  | FOV (9.6, 15, 26, 36cm) |  | | N |  |  |  |  |
| (110) | Vogel et al. | 2008 | 80 | Patients undergoing staging CT for malignancy | 64CT | Bland-Altman (%) | volume | slice thickness (1, 3, 5mm) | smallest at 3mm | | N |  | | | |
| (111) | Wang et al. | 2008 | 4225 | LCS participants | 16CT, LDCT | Bland-Altman (mm3, %) | interobserver variability |  | [-13.4%,14.0%] | |  |  | | | |
|  |  |  |  |  |  | Multivariate logistic regression | measurement discrepancy | location (pleural based, attached to fissure, intra-parenchymal, juxtavascular) | ↑ | | Y |  | | | |
|  |  |  |  |  |  |  |  | shape (polylobulated, spiculated, smooth, irregular) | ↑ | | Y |  | | | |
|  |  |  |  |  |  |  |  | nodule size (≤50mm3, >50mm3) | ↓ | | Y |  | | | |
| (112) | Way et al. | 2008 | 41 | synthetic nodules (diameter: 4.8, 9.5, 16mm) | 64CT | Student t-test ANOVA | Accuracy (APE) | slice thickness (0.625, 2.5mm) | ↓(↑) | | Y |  | |  |  |
|  |  |  |  |  |  |  |  | pitch (0.531:1 - 1.375:1) |  | | N |  |  |  |  |
|  |  |  |  |  |  |  |  | FOV (25 - 36cm) |  | | N |  |  |  |  |
|  |  |  |  |  |  |  |  | tube current (80 - 400mAs) |  | | N |  |  |  |  |
| (113) | Biederer et al. | 2007 | 55 | synthetic nodules (mean diameter: 8.4mm ± 1.8) | 4DCT 40CT | Wilcoxon matched-pairs test | volume | level of inspiration | ↑ (end-respiratory) | | Y |  | |  |  |
|  |  |  |  |  |  |  |  | scan (static, dynamic) |  | | N |  | |  |  |
|  |  |  |  |  |  |  | variation coefficient | scan (static, dynamic) | ↑ | | Y |  | |  |  |
|  |  |  |  |  |  |  |  | nodule size |  | | N |  | |  |  |
|  |  |  |  |  |  |  |  | nodule displacement during breathing |  | | N |  | |  |  |
|  |  |  |  |  |  |  |  | caudal location | ↑ | | Y |  | |  |  |
| (114) | Bolte et al. | 2007 | 46 | synthetic nodule (fat-wax-Lipiodol, density: 50-150HU) | 16CT, SDCT | Wilcoxon signed-rank test | variation coefficient | training effect (untrained, trained) | ↓ | | Y |  | |  |  |
|  |  |  |  |  |  |  |  | observer (unexperienced, experienced) |  | | N |  | |  |  |
| (115) | Bolte et al. | 2007 | 20 | synthetic nodules (fat-wax-Lipiodol mixture) | 16CT | Ansari-Bradley test | intrascan | CT protocol (routine thoracic RTP, special volumetric protocol SVP) | RTP [-3.1%,4.3%] SVP [0,1.5%] | | Y |  | |  |  |
|  |  |  |  |  |  |  | interscan |  | RTP [-7.0%,3.5%] SVP [-10.8%,2.9%] | | N |  | |  |  |
|  |  |  |  |  |  |  | interobserver |  | RTP [-0.4%,6.8%] SVP [-4.3%,3.3%] | | Y |  | |  |  |
| (116) | Bolte et al. | 2007 | 202 | synthetic nodule (fat-wax-Lipiodol, density: 50-150HU) | 16CT | ANOVA | volume error (%) | semi-automatic | size: all [-29.9%,11.6%] size: 5-10mm [-30.1%,12.7%] size: ≥10mm [-28.2%,-2.4%] | |  |  | |  |  |
|  |  |  |  |  |  |  |  | manually corrected | size: all [-13.0%,12.4%] size: 5-10mm [-13.8%,13.2%] size: ≥10mm [-7.9%,7.3%] | |  |  | |  |  |
|  |  |  |  |  |  |  |  | semi-automatic vs manually corrected | ≠ (5-10mm) | | Y |  | |  |  |
| (117) | Das et al. | 2007 | 40 | synthetic nodules (location: intraparenchymal, juxtavascular, attached to vessel, juxtapleural, attached to pleura; diameter: 3-10mm) | 16CT | Three-way ANOVA | Accuracy (APE) | CT scanner vendors (Siemens, GE, Philips, Toshiba) |  | | Y |  | |  |  |
|  |  |  |  |  |  |  |  | radiation dose exposure (SDCT, LDCT) |  | | N |  | |  |  |
|  |  |  |  |  |  |  |  | tube current (20, 100mAs) |  | | N |  | |  |  |
|  |  |  |  |  |  |  |  | collimation (thin, thick) |  | | Y |  | |  |  |
|  |  |  |  |  |  |  |  | slice thickness |  | | Y |  | |  |  |
|  |  |  |  |  |  |  |  | nodule size | ↑(↓) (5-10mm) | |  |  | |  |  |
|  |  |  |  |  |  |  |  |  |  | |  |  | | | |
| (118) | Gietema et al. | 2007 | 218 | Patients with lung metastases | 16CT, LDCT | Bland-Altman (%) | interscan variability |  | [-21.2%,23.8%] | |  |  | | | |
|  |  |  |  |  |  | ANOVA |  | segmentation (complete, incomplete) | complete [-11.9%,12.4%] incomplete [-26.8%,30.0%] | | Y |  |  |  |  |
|  |  |  |  |  |  | Univariate linear regression | volume | level of inspiration |  | | N |  |  |  |  |
| (119) | Honda et al. | 2007 | 60 | Patients with lung nodules <3cm | 64CT | Wilcoxon signed-rank test | volume | kernel (standard, bone) | ↑ | | Y |  | | | |
|  |  |  |  |  |  |  |  | contrast enhancement (pre-, post-contrast) | ↑ | | Y |  | | | |
| (120) | Obenauer et al. | 2007 | 4 | synthetic nodules (diameter: 3, 3.18, 3.97, shape: spherical; material: nylon, teflon, polypropylene); (shape: irregular) | fpVCT, 64CT | repeated-measures ANOVA | volume error (mm3) | segmentation algorithm (region growing, threshold, planimetry, ellipsoid, single advanced lung analysis [ALA]) | ≠ | | Y |  | |  |  |
|  |  |  |  |  |  |  |  | scanner (fpVCT, 64CT) | ↓ | | Y |  | |  |  |
| (121) | Petkovska et al. | 2007 | 75 | Emphysema related trial participants | 6CT, 16CT, 64CT | Linear regression | absolute volume change | level of inspiration | ↓ | | Y |  | | | |
| (122) | Bolte et al. | 2006 | 70 | synthetic nodules (density: 50 - 150HU) | 16CT | Variation coefficient | intrascan reproducibility | volumetry tool / human | 6.2% / 0.7% | |  |  | |  |  |
|  |  |  |  |  |  |  | interscan reproducibility |  | 9.2% / 3.7% | |  |  | |  |  |
|  |  |  |  |  |  | Kruskal-Wallis test | intrascan variation coefficient | nodule size |  | | Y |  | |  |  |
| (123) | Das et al. | 2006 | 40 | synthetic nodules (diameter: 3-10mm; location: intraparenchymal, around vessel, attached to vessel, around pleura, attached to pleura) | SSCT, 4CT, 16CT, 64CT | ANOVA | Accuracy (APE) | radiation dose exposure (SDCT, LDCT) |  | | N |  | |  |  |
|  |  |  |  |  |  |  |  | collimation (thin, thick) |  | | Y |  | |  |  |
|  |  |  |  |  |  |  |  | location |  | | Y |  | |  |  |
|  |  |  |  |  |  |  |  | nodule size | ↑(↓) | | Y |  | |  |  |
|  |  |  |  |  |  |  |  | scanner detectors | (4CT vs 64CT and 16CT vs 64CT) | | Y |  | |  |  |
| (124) | Gietema et al. | 2006 | 430 | LCS participants with solid intraparenchymal, non-calcified nodules | 16CT, LDCT | Spearman correlation coefficient | interobserver correlation | nodule size | ↓ | | Y |  | | | |
|  |  |  |  |  |  |  |  | shape (smooth, polylobulated, irregular, spiculated) | ↓ (irregular) | | Y |  | | | |
|  |  |  |  |  |  |  |  | form (round, oval, elliptic) |  | | N |  | | | |
|  |  |  |  |  |  | Bland-Altman (mm3, %) | interobserver variability |  | all [-8.2%,9%] discrepancies [-21.9%,28.5%] | |  |  | | | |
| (125) | Goo et al. | 2006 | 33 | Patients with severe asthma | 16CT, SDCT | paired t-test | volume | level of inspiration (inspiration, expiration) | ↑ | | Y |  | | | |
|  |  |  |  |  |  | Linear correlation analysis | Accuracy (APE) | density |  | | N |  | | | |
|  |  |  |  |  |  |  |  | total lung volume | (diameter ≥3mm) | | Y |  | | | |
|  |  |  |  |  |  |  |  | whole lung attenuation |  | | N |  | | | |
|  |  |  |  |  |  |  |  | attenuation of adjacent lung parenchyma | ↑(↓) | | Y |  | | | |
| (126) | Honda et al. | 2006 | 39 | 50 nonconsecutive patients with solid peripheral non-calcified and non-cavitating pulmonary nodules | 8CT | Kruskal-Wallis test Wilcoxon signed-rank test | volume | slice thickness (1.25, 2.5, 3.75, 5mm) | ↑ (5mm) | | N |  | | | |
|  |  |  |  |  |  |  |  | FOV (10, 20, 30cm) | ↑ (20cm) | | N |  | | | |
|  |  |  |  |  |  |  |  | kernel (high-spatial, low-spatial frequency) | ↓ | | Y |  | | | |
|  |  |  |  |  |  |  |  | overlap (0%, 50%) | ↓ | | Y |  | | | |
| (127) | Kuhnigk et al. | 2006 | 42 | 3 software nodules (diameter: 3, 6, 9mm; shape: spherical; density: 50HU) + 39 synthetic nodules (shape: spherical, nonspherical, location: intra-parenchymal, juxtavascular, juxtapleural, diameter: 3 - 10mm) | 64CT | plot | % volume change | threshold | ≠ (conventional segmentation algorithm) | | Y |  | |  |  |
|  |  |  |  |  |  | Bland-Altman (%) | inter-algorithm variability | tube current (20, 120mAs) | CONV algorithm [-1.5%,11.1%] SPVA algorithm [-1.9%,9.6%] | |  |  | |  |  |
|  |  |  |  |  |  |  |  | slice thickness (0.6, 1, 2mm) | CONV algorithm [-31.9%.1.6%] SPVA algorithm [-19.7%,3.1%] | |  |  | |  |  |
|  |  |  |  |  |  |  |  | kernel (B46f, B50f, B60f) | CONV algorithm [-30.5%.-1.8%] SPVA algorithm [-8.4%,4.6%] | |  |  | |  |  |
|  |  |  | 8 | Patients with lung metastases | 4CT | Bland-Altman (mm3, %) | interobserver variability |  | [-7.1%,7.1%] | |  |  | | | |
|  |  |  |  |  |  |  | interscan variability |  | [-27%,27%] | |  |  | | | |
| (128) | Petrou et al. | 2006 | 75 | Patients with pulmonary nodules between 3-20mm, solid, intra-parenchymal | 16CT LDCT | Student t-test | Accuracy (APE) | slice thickness / interval (1.25/0.625, 2.5/2, 5/2.5mm) | ↑(↓) (small nodules) | | Y |  | | | |
|  |  |  | 69 |  |  | Multiple linear regression | volume variability | slice thickness (1.25, 2.5mm) |  | | Y |  |  |  |  |
|  |  |  |  |  |  |  |  | shape (smooth, spiculated) | ↑ | | Y |  |  |  |  |
|  |  |  |  |  |  |  |  | software | ≠ (2.5mm slice thickness) | | Y |  |  |  |  |
| (129) | Picozzi et al. | 2006 | 20 | synthetic nodules (diameter: 8mm, density: 115HU, shape: irregular) + (diameter: 8mm, location: adjacent to pulmonary structures) + (diameter: 8.3mm; density: 50HU, deformable) | SSCT, 4CT | Wilcoxon matched-pairs test | observer-dependent reproducibility | scanner (SSCT, 4CT) |  | | N |  | |  |  |
|  |  |  |  |  |  | Kendal τ test |  | observer-dependent repeatability |  | | N |  | |  |  |
|  |  |  | 37 | LCS participants with solid nodules ≥5mm |  | Kendal τ test |  | observer-dependent repeatability |  | | Y |  | | | |
|  |  |  |  |  |  | Bland-Altman | intraobserver variability |  | phantom [-3%,3%] in vivo [-3.1%,2.8%] | |  |  | | | |
| (130) | Reeves et al. | 2006 | 50 | *In vivo* screening database | SDCT |  | percent volume change | segmentation algorithm | ≠ | | Y |  | | | |
| (131) | Volterrani et al. | 2006 | 103 | Patients with solid, noncalcified nodules, with peripheral ground-glass attenuation and central cavitation | 16CT | ANOVA | volume reproducibility | location (intra-parenchymal, juxtavascular, juxtapleural) | ↓ | | Y |  | | | |
|  |  |  |  |  |  |  |  | nodule size |  | | N |  | | | |
| (132) | Goo et al. | 2005 | 720 | synthetic nodules (diameter: 3.2, 4.8, 6.4, 12.7mm) | 16CT, LSDCT | MANOVA | Accuracy (APE) | slice thickness (0.75, 1, 2, 3, 5mm) |  | | Y | threshold * slice thickness | |  |  |
|  |  |  |  |  |  |  |  | threshold (-600, -500, -400, -300HU) |  | | Y |  |  |  |  |
|  |  |  |  |  |  |  |  | FOV (10, 20, 30cm) |  | | N |  |  |  |  |
|  |  |  |  |  |  |  |  | reconstruction internal (0.5, 1, 2mm) |  | | N |  |  |  |  |
|  |  |  |  |  |  | Regression |  | nodule size | ↑(↓) | | Y |  |  |  |  |
| (133) | Ko et al. | 2005 | 51 | LCS participants | LDCT | mixed-model ANOVA | volume | image compression (10:1, 20:1, 30:1, 40:1) | ↓ (images compressed to ≥20:1) (more significant in subsolid nodules) | | Y | compression * nodule size, compression * location, compression * density | | | |
|  |  |  |  |  |  |  | Accuracy (APE) | nodule size (≤5, >5mm) | ↑(↓) | | Y |  |  |  |  |
|  |  |  |  |  |  |  |  | location (central, peripheral, juxtapleura, juxtavascular) |  | | Y |  |  |  |  |
|  |  |  |  |  |  |  |  | density | ↑(↓) | | Y |  |  |  |  |
| (134) | Boll et al. | 2004 | 30 | synthetic nodules (shape: spherical) |  | one-way ANOVA |  | cardiac phase |  | | N |  | |  |  |
|  |  |  | 73 | Patients who underwent ECG-gated CT | 16CT | Pillai-Bartlett trace Wilks lambda test | volume variation | cardiac phase |  | | Y |  | | | |
|  |  |  |  |  |  |  |  | location (pulmonary lobe segments) | ↑ (segments close to the heart and aorta) | | Y |  | | | |
|  |  |  |  |  |  |  |  | density |  | | N |  | | | |
|  |  |  |  |  |  | logistic regression |  | nodule size | ↓ | | Y |  | | | |
| (135) | Goodman et al. | 2004 | 43 | Patients with pulmonary nodules <20mm | 8CT, 16CT, SDCT | Bland-Altman (%) | interobserver variability |  | [-0.018%,0.018%] | |  |  | | | |
|  |  |  |  |  |  |  | interscan variability |  | [-25.6%,25.6%] | |  |  | | | |
|  |  |  |  |  |  |  |  | diameter | <6mm [-27.6%,27.6%] 6 - <9mm [-31.4%,31.4%] ≥9mm [-17.9%,17.9%] | |  |  | | | |
|  |  |  |  |  |  |  |  | density | solid [-24.4%,24.4%] partly calcified [-23.6%,23.6%] totally calcified [-22.7%,22.7%] | |  |  | | | |
|  |  |  |  |  |  |  |  | contrast | enhanced [-32.7%,32.7%] unenhanced [-24.8%,24.8%] | |  |  | | | |
|  |  |  |  |  |  |  |  | margins | smooth/lobulated [-26.4%,26.4%] irregular/spiculated [-25.2%,25.2%] | |  |  | | | |
|  |  |  |  |  |  |  |  | adjacent parenchyma | normal [-25.6%,25.6%] abnormal [-27.1%,27.1%] | |  |  | | | |
|  |  |  |  |  |  |  |  | calcified or enhanced | yes [-28.4%,24.8%] no [-24.8%,24.8%] | |  |  | | | |
| (136) | Kostis et al. | 2004 | 115 | LCS participants with stable nodules | SSCT, MDCT, SDCT | mean, SD | percent volume change | nodule size | ↓ | |  |  | | | |
|  |  |  |  |  |  |  |  | motion artifacts (absent, present) | ↑ | |  |  | | | |
| (137) | Marten et al. | 2004 | 70 | synthetic nodules (diameter: 10, 12mm); density: +100, -630, -800HU) | fpVCT, 4CT | Wilcoxon signed-rank test | Accuracy (APE) | scanner (fpVCT, 4CT) | ↓(↑) | | Y |  | |  |  |
|  |  |  |  |  |  |  |  | collimation (0.63, 1.25) | ↓(↑) | | Y |  | |  |  |
|  |  |  |  |  |  |  |  | nodule size | ↑(↓) | | Y |  | |  |  |
| (138) | Marten et al. | 2004 | 50 | synthetic nodules (diameter: 1.36-5.34mm) | fpVCT | Wilcoxon signed-rank test | Accuracy (APE) | deformation | ↓(↑) | | Y |  | |  |  |
|  |  |  |  |  |  |  |  | nodule size | ↑(↓) | | Y |  | |  |  |
| (139) | Mullaly et al. | 2004 | 40 | synthetic nodules (diameter: 2.4, 3.2, 4, 4.9mm) shape: spherical, density: -360HU, 50HU | 4CT | Sum of squared errors | Accuracy (APE) | nodule size | ↑(↓) | | Y |  | |  |  |
|  |  |  |  |  |  |  |  | density (ground-glass, solid) | ↑(↓) | | Y |  |  |  |  |
|  |  |  |  |  |  |  |  | segmentation algorithm |  | | N |  |  |  |  |
|  |  |  |  |  |  |  |  | kernel |  | | N |  |  |  |  |
|  |  |  | 29 | Patients with chest CT for clinical indications |  | Sum of squared errors | Accuracy (APE) | segmentation algoritgm | ≠ | | Y |  | | | |
| (140) | Revel et al. | 2004 | 52 | Patients with solid, noncalcified pulmonary nodules between 5 - 18mm | 4CT | Bland-Altman (%) | intraobserver variability |  | R1 [-8.9%,8.9%] R3 [-7.6%,7.6%] | |  |  | | | |
|  |  |  |  |  |  | pooling of measurements | global repeatability of volume |  | [-6.38%,6.38%] | |  |  | | | |
| (141) | Wormanns et al. | 2004 | 151 | Patients with lung metastases | LDCT 4CT | Bland-Altman (%) | intraobserver |  | [-3.9%,5.7%] | |  |  | | | |
|  |  |  |  |  |  |  | interobserver |  | [-5.5%,6.6%] | |  |  | | | |
|  |  |  |  |  |  |  | intrascan |  | [-20.4%,21.9%] | |  |  | | | |
|  |  |  |  |  |  |  | intrascan | nodule size <10mm | [-19.3%,20.4%] | |  |  | | | |
| (142) | Ko et al. | 2003 | 40 | synthetic nodules (diameter: 2 - 5mm, density: -360, 50HU; shape: spherical) | 4CT, LDCT, SDCT | ANOVA | Accuracy (APE) | tube current (20, 120 mAs) | ↑(↓) | | Y | segmentation algorithm * nodule size, segmentation algorithm * tube current, segmentation algorithm * density | |  |  |
|  |  |  |  |  |  |  |  | density (ground-glass, solid) | ↑(↓) | | Y |  |  |  |  |
|  |  |  |  |  |  |  |  | nodule size | ↑(↓) | | Y |  |  |  |  |
|  |  |  |  |  |  |  |  | segmentation algorithm (partial volume, threshold method) | ↓(↑) (small nodules) | | Y |  |  |  |  |
|  |  |  |  |  |  |  |  | location |  | | N |  |  |  |  |
| (143) | Yankelevitz et al. | 2000 | 21 + 20 + 35 | synthetic nodules (diameter: 3.2, 3.96mm, shape: spherical) + (nodules before and after deformation) + (nodules of various shapes and sizes) | SSCT, SDCT | Coefficient of variation | Accuracy (APE) | nodule size | ↓(↑) (small nodules) < 3% | |  |  | |  |  |
|  |  |  | 13 | Patients with pulmonary nodules <10mm | SSCT, SDCT | Coefficient of variation | Accuracy (APE) | nodule size | ↓(↑) (small nodules) < 3% | |  |  | | | |

**Supplementary Table 2.** Studies that seemed promising for inclusion during the screening phase but were later excluded.

| Ref. | Authors | Year | Design | Outcome |
| --- | --- | --- | --- | --- |
| (144) | Ahn et al. | 2022 | *in vivo* | Recurrence-free survival after metastasectomy |
| (145) | Hammer et al. | 2021 | *in vivo* | Sensitivity and specificity for lung cancer |
| (146) | Solomon et al. | 2020 | phantom | Visual detection of growth |
| (147) | dos Santos et al. | 2020 | *in vivo* | Pulmonary nodule detection |
| (148) | Hwang et al. | 2020 | *in vivo* | Risk of lung cancer within one year |
| (149) | Silva et al. | 2020 | *in vivo* | Risk of lung cancer |
| (150) | Setojima et al. | 2019 | *in vivo* | Recurrence-free survival |
| (151) | Kamiya et al. | 2018 | *in vivo* | Post-operative recurrence and prognosis |
| (152) | Li et al. | 2018 | *in vivo* | Risk of invasiveness of adenocarcinoma |
| (153) | Mets et al. | 2018 | *in vivo* | Growth pattern |
| (154) | Sun et al. | 2018 | *in vivo* | VDT |
| (155) | Heuvelmans et al. | 2017 | *in vivo* | Growth pattern |
| (156) | Heuvelmans et al. | 2017 | *in vivo* | Axial diameter-based volume overestimation |
| (157) | Gavrielides et al. | 2016 | phantom | Detection of volume change |
| (158) | Hwang et al. | 2016 | *in vivo* | Sensitivity and specificity for lung metastases |
| (159) | Li et al. | 2016 | *in vivo* | Positive cases I-ELCAP |
| (160) | Li et al. | 2016 | *in vivo* | Risk of malignancy |
| (3) | Dicken et al. | 2015 | *in vivo* | Diameter of a volume equivalent sphere |
| (161) | Shen et al. | 2015 | *in vivo* | Whole lung volume over-segmentation |
| (162) | Smith et al. | 2015 | *in vivo* | Error in growth rate |
| (163) | Horeweg et al. | 2014 | *in vivo* | Sensitivity and specificity for lung cancer |
| (1) | Horeweg et al. | 2014 | *in vivo* | Risk of lung cancer within two years |
| (164) | Shin et al. | 2014 | *in vivo* | Risk of malignancy |
| (165) | Yanagawa et al. | 2014 | *in vivo* | Disease-free survival / Overall survival |
| (166) | Horeweg et al. | 2013 | *in vivo* | Risk of malignancy |
| (167) | Stolten et al. | 2013 | *In vivo* | Comparing volumetry with manual segmentation |
| (168) | Henschkle et al. | 2012 | *in vivo* | VDT |
| (169) | Kim et al. | 2012 | *in vivo* | VDT |
| (170) | Tanimoto et al. | 2012 | *in vivo* | VDT |
| (171) | Vogel et al. | 2012 | *in vivo* | Response type |
| (172) | Ko et al. | 2011 | *in vivo* | Growth rate |
| (5) | Korst et al. | 2011 | *in vivo* | Growth rate |
| (173) | van Klaveren et al. | 2009 | *in vivo* | Risk of malignancy |
| (174) | Pauls et al. | 2008 | *in vivo* | Diameter, area |
| (175) | Revel et al. | 2005 | *in vivo* | Risk of malignancy |

**Supplementary Table 3:** Results of the attempted meta-analysis of two sets of similar studies.

|  | bias | _lower_ LOA | _upper_ LOA | _lower_ CI_m_ | _upper_ CI_m_ | _lower_ CI_rv_ | _upper_ CI_rv_ | 𝜏^2^ |
| --- | --- | --- | --- | --- | --- | --- | --- | --- |
| SDCT vs ULDCT (95,105) | 1,67% | -26,22% | 29,56% | -36,56% | 39,89% | -36,55% | 39,89% | -2,19E-05 |
| Patients with pulmonary metastases (8,118,141) | 0,36% | -19,77% | 20,49% | -29,21% | 29,93% | -29,88% | 30,60% | 7,14E-05 |

_lower_ CI_m_: 95% Confidence interval (_95_CI) of the lower LOA using a model-based estimator; _upper_ CI_m_: _95_CI of the upper LOA using a model-based estimator; _lower_ CI_rv_: _95_CI of the lower LOA using robust variance estimation (RVE); _upper_ CI_rv_: _95_CI of the upper LOA using RVE; 𝜏^2^: estimator for between-study variation in bias in the LOA.

**Supplementary Figure 1**


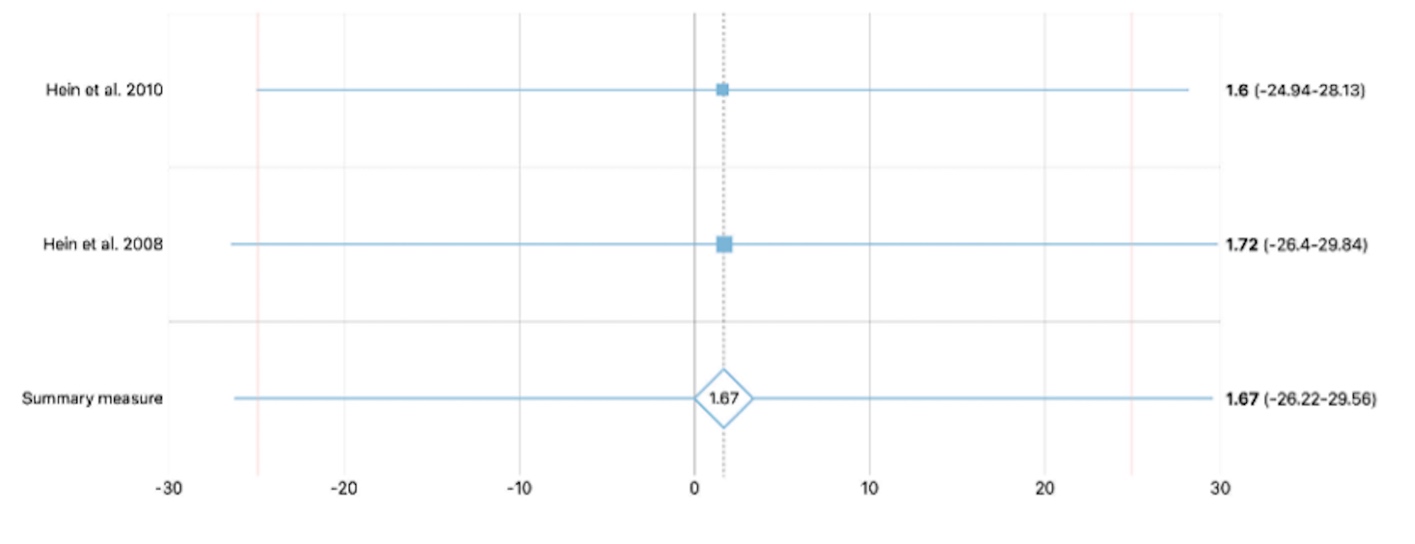


**
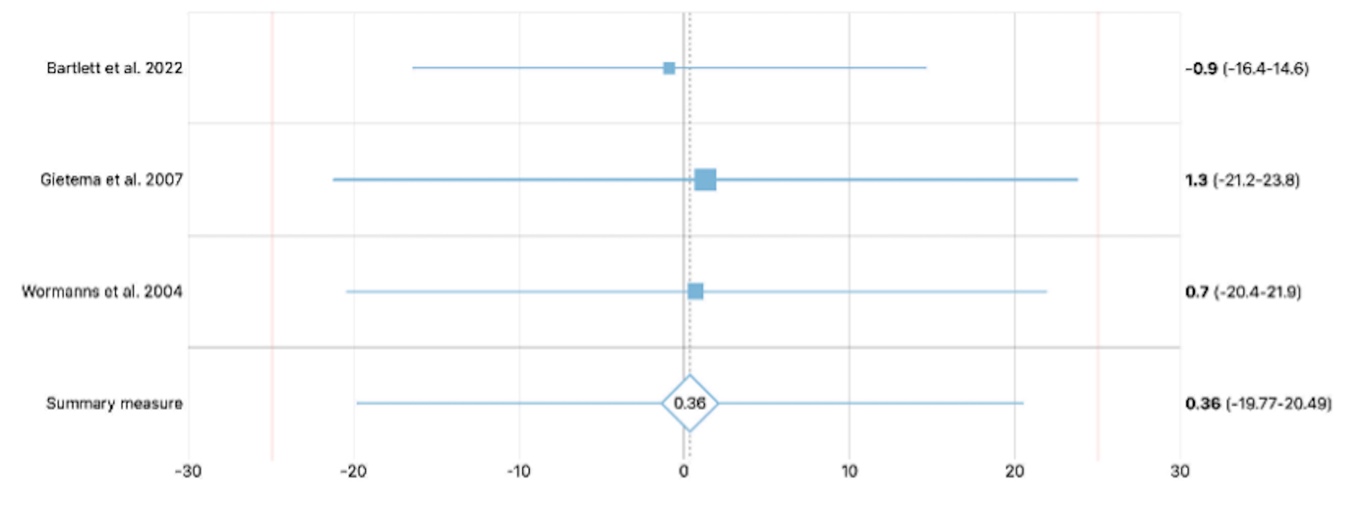
Supplementary Figure 2**
